# A Review on Mycotoxins and Microfungi in Spices in the Light of the Last Five Years

**DOI:** 10.3390/toxins12120789

**Published:** 2020-12-11

**Authors:** Darina Pickova, Vladimir Ostry, Jan Malir, Jakub Toman, Frantisek Malir

**Affiliations:** 1 Department of Biology, Faculty of Science, University of Hradec Kralove, Rokitanskeho 62, CZ-50003 Hradec Kralove, Czech Republic; ostry@chpr.szu.cz (V.O.); jakub.toman@uhk.cz (J.T.); frantisek.malir@uhk.cz (F.M.); 2 Center for Health, Nutrition and Food in Brno, National Institute of Public Health in Prague, Palackeho 3a, CZ-61242 Brno, Czech Republic; 3 Department of Public Law, Institute of State and Law, Czech Academy of Sciences, Narodni 18, CZ-11600 Prague, Czech Republic; jan.malir@ilaw.cas.cz

**Keywords:** spices, contamination, microfungi, mycotoxin

## Abstract

Spices are imported worldwide mainly from developing countries with tropical and/or subtropical climate. Local conditions, such as high temperature, heavy rainfall, and humidity, promote fungal growth leading to increased occurrence of mycotoxins in spices. Moreover, the lack of good agricultural practice (GAP), good manufacturing practice (GMP), and good hygienic practice (GHP) in developing countries are of great concern. This review summarizes recent data from a total of 56 original papers dealing with mycotoxins and microfungi in various spices in the last five years. A total of 38 kinds of spices, 17 mycotoxins, and 14 microfungi are discussed in the review. Worldwide, spices are rather overlooked in terms of mycotoxin regulations, which usually only cover aflatoxins (AFs) and ochratoxin A (OTA). In this paper, an extensive attention is devoted to the limits on mycotoxins in spices in the context of the European Union (EU) as well as other countries. As proven in this review, the incidence of AFs and OTA, as well as other mycotoxins, is relatively high in many spices; thus, the preparation of new regulation limits is advisable.

## 1. Introduction

The attention of the professional public has been focused on systematic control of the presence of xenogenous substances in foodstuffs which might endanger the health state of the population. This is also the case of mycotoxins, toxic secondary metabolites of microfungi. Specific problems and risks arise from the global climate change and globalization of the food market—these processes may result in an increased occurrence of mycotoxins due to many reasons including the extension of the scale of foodstuffs from regional sources, changes of food storage, transportation or dietary patterns [1,2]. Spices have been widely used since ancient times and that, primarily, for their unique flavoring, coloring, and aromatizing properties and, secondarily, for preservative, antimicrobial, and antioxidant effects. Moreover, their beneficial effect on human health is valued both in traditional and modern medicine [3,4]. One of the definitions describes spices as non-leafy parts of a plant such as bud, fruit, seed, bark, rhizome or bulb; parts derived from leaf or flower of a plant are considered to form a distinct group—herbs [5]. However, all parts of a plant should be considered to be spices if they possess the aforementioned properties for meal enhancement, such as its color, flavor, or even texture [4]. In this review, spices have been selected in line with the definition by Uhl [4] and at the discretion of the authors.

Unfortunately, certain spices are very susceptible to toxigenic microfungi growth and thus potential mycotoxin development [3,6,7,8]. It is known that “spices” are generally more susceptible to contamination than “herbs” [9,10]. Moreover, spices purchased in open markets are confirmed to be significantly more contaminated than spices purchased in supermarkets [11].

Agricultural land with infected plant residues serves as the main reservoir of microfungi. Agricultural products can be infected with spores in situ or ex situ via dust or insects [12]. The mycotoxin contamination of agricultural commodities is a common phenomenon and despite of various prevention technologies and recommendations cannot be completely avoided [7]. However, some preventing physical, chemical, and biological strategies have been developed [7,13,14]. Nevertheless, in the EU, chemical treatments are not allowed for the decontamination of foodstuffs [15]. Appropriate and well-designed strategies could result in the reduction of mycotoxins in spices [7]. Beside innovative technologies, following GAP, GMP, and GHP is also necessary to prevent mold growth and mycotoxin production [7]. Inappropriate conditions during pre-harvest, harvest, and post-harvest can affect the quality of the spices. Good hygienic and physical separation are the best approaches for mycotoxin management in spices [7]. Maintaining good practices can, however, be problematic as spices are mainly grown in the developing countries from where they are exported and distributed worldwide. Moreover, their contamination is further supported by local subtropical and tropical climate characterized by high temperatures, heavy rainfalls, and relative humidity providing suitable conditions for fungal growth and thus mycotoxin production [1,2,16,17]. Fungal growth is also affected by the landform, soil types, and its properties, as well as interactions between the microfungus and micro- and macro-organisms in the soil [18,19]. Mycotoxins in the soil can be absorbed by plant roots and transported via the xylem to plant tissues [20].

This review summarizes only recent relevant original papers published in the last five years (since 2015). We consider this time span to be appropriate in terms of reflecting the current situation. A fair deal of studies concerning mycotoxins and/or microfungi in spices has been published. In the last five years, a total of 147 and 127 papers dealing with “mycotoxins” and “fungi” in “spices” have been found in the Web of Science database and a total of 52 and 45 publications in PubMed database, respectively. In total, 56 relevant papers were selected as the basis for this review. The quality criteria for the comparative analysis of individual studies were validation of analytical methods and quality of analytical results of mycotoxin determination.

## 2. Spices as a Part of the Worldwide Diet

Spices, as an essential part of the human diet, are normally used in small amounts for food flavoring [21]. Spice consumption varies worldwide, depending on the country and local eating habits [22]; however, there is a limited number of scientific publications concerning spice consumption providing comprehensive data on its intake into the human body.

As for European and American countries, oregano is considered the most consumed herbal spice, followed by basil, bay leaf, parsley, thyme, and chives [22]. In the recent study, pepper, paprika, parsley, and basil were labeled the most commonly used spices in the European Union (EU) [23]. 

As for Asia, commonly used spices include black pepper, cardamom, cinnamon, cassia, chili pepper, cloves, coriander, cumin, garlic, ginger, nutmeg, mace, turmeric, and vanilla [5]. Chili pepper is the most commonly used spice in India, consumed in much higher amounts per portion than other spices. Based on the total amount of consumed spice (amount per portion and frequency of consumption), chili pepper (on average 3.0 g per portion), cumin (1.64 g), turmeric (0.71 g), coriander (1.37 g) and mustard (1.07 g) can be considered the top five most important spices in India. Caraway, cinnamon, cardamom, cloves, black pepper, garlic, and ginger are also commonly used in India [21,24]. Less used are asafetida, carom, mace, and nutmeg [21]. Fenugreek is also among the less important spices in India [21]; however, apart from its use as a spice, people also consume its seeds as food [25]. In China, commonly used spices and herbs include garlic, onions, chili pepper, coriander, basil, cinnamon, star anise, and ginger [26,27]. In addition, some herbs and spices are used in traditional Chinese medicine, e.g., galangal or nutmeg [28]. In Thailand, chili pepper, onion (shallot), and garlic are the most used spices. Other common spices include lemongrass, galangal, basil, mint, and fennel [29].

As for African countries, many commonly used spices are world-known such as garlic, ginger, chili pepper (*Capsicum frutescens*), onion, nutmeg or pepper (Ashanti pepper, *Piper guineese*) [30,31,32,33], while some spices are typical for Africa, such as, e.g., dawadawa, ogiri, okpehe, hwentia, soro wisa or fem wisa [31,34]. Based on a study by Nguegwouo et al. [35], cloves, white pepper, and black pepper are also common in Africa. The daily intake of white pepper (mean 1.924 g) is approximately two times higher than the daily intake of black pepper (mean 0.939 g) in Cameroon [35].

As evident, chili pepper (*Capsicum* spp.) and peppers (*Piper* spp.) are ubiquitous spices, normally consumed in quantities of a few grams per day in many places around the world. Moreover, garlic and onion (*Allium* spp.) can be considered to be one of the most used spices worldwide [36]. This makes *Capsicum* spp., *Piper* spp. and *Allium* spp. one of the most important spices from the perspective of xenogenous substance and thus also mycotoxin studies. However, many other world-known spices as well as local and traditional spices are also consumed in relatively high amounts and should be taken into consideration.

## 3. The Worldwide Spice Production

**Figure 1:**
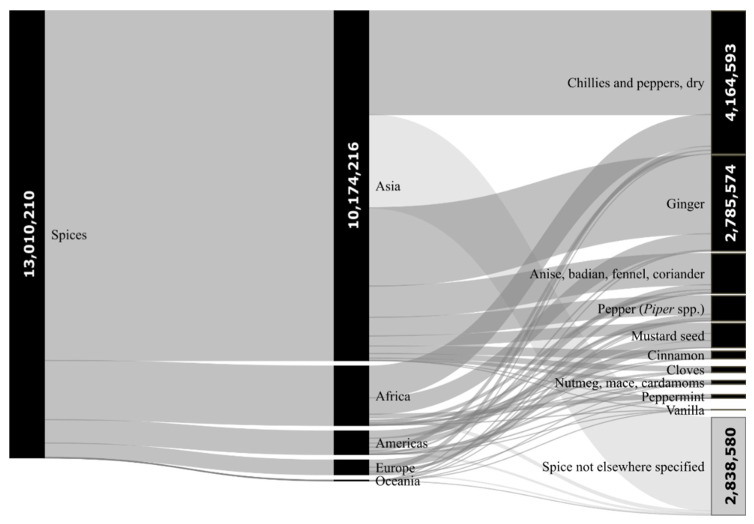
The share of spice production in tonnes in the last available year 2018. Note: Number of tonnes produced in brackets: Africa (1,722,909); Americas (679,830); Europe (420,188); Oceania (13,067); Anise, badian, fennel, coriander (1,165,683); Pepper, *Piper* spp. (732,523); Mustard seed (710,350); Cinnamon (221,815); Cloves (167,506); Nutmeg, mace, cardamoms (109,284); Peppermint (106,728); Vanilla (7574). Processed according to FAOSTAT [37].

According to the data available over the last 5 years (the latest available data from the years 2014–2018), the average worldwide production of spices was c. 12.3 million tonnes per year (13.0 million tonnes in 2018) and consisted especially of the following spices: “Anise, badian, fennel, coriander”, “Chilies and peppers, dry” “Cinnamon”, “Cloves”, “Ginger”, “Nutmeg, mace, cardamoms”, “Mustard seed”, “Pepper, *Piper* spp.”,”Peppermint”, “Vanilla” and “Spice not elsewhere specified”. The items such as “Garlic” and “Onions dry” were not included, as their production of 27.8 million tonnes and 84.3 million tonnes, respectively (2018), would increase the total spice production approximately ten times. Asia, with its production share of 78.2% (10.2 million tonnes in 2018), is undoubtedly the largest producer of spices in the present world—see Figure 1. India contributes most to this share (5.4 million tonnes in 2018), by far followed by China (1.2 million tonnes in 2018) [37]. Top 20 world producers are shown in Table 1.

## 4. Characterization of Mycotoxins and Their Producers Included in This Review

Mycotoxins, one of the most serious contaminants of natural origin [38], are produced by toxigenic microfungi, mostly by *Aspergillus*, *Penicillium*, and *Fusarium* [8,39] and to a certain extent *Alternaria* genera [40] as their secondary metabolites [38,41]. Moreover, some highly mycotoxigenic microfungi have not been described yet [42].

Currently, more than 500 mycotoxins have been identified, but only a few of them normally occur in the human diet in significant amounts and, consequently, pose a potential threat to human and/or animal health [43]. AFs, OTA, fumonisins (FMNs), zearalenone (ZEA), citrinin (CIT), and trichothecenes (TCT)—deoxynivalenol (DON) and nivalenol (NIV) are considered to be some of the most important in terms of toxic effect and high prevalence in the agro-food commodities [44,45], including spices [44]. In addition, *Alternaria* mycotoxins are also common contaminants in spices and other agricultural products [46]. Mycotoxins dealt with in this review are listed below; their chemical structures are shown in Table 2.

### 4.1. Aflatoxins

AFs are the world´s most studied mycotoxins [48] as they have been shown to have hepatotoxic, genotoxic, mutagenic, teratogenic, immunosuppressive, nephrotoxic, cytotoxic, and mainly carcinogenic effects [49,50,51]. According to the International Agency for Research on Cancer (IARC), all mentioned AFs are classified into group 1 “*Carcinogenic to humans*” [52].

The most common AFs include aflatoxin B_1_ (AFB_1_) (PubChem CID: 186907), aflatoxin B_2_ (AFB_2_) (PubChem CID: 2724360), aflatoxin G_1_ (AFG_1_) (PubChem CID: 14421) and aflatoxin G_2_ (AFG_2_) (PubChem CID: 2724362) [47]. AFB_1_ is thought to be the most significant [50].

AFs are produced by *Aspergillus* species, mainly by *A. flavus*, and *A. parasiticus* [53,54,55]. *A. nomius* [55] and *A. pseudotamarii* have also been reported to be aflatoxigenic in food [42,56].

### 4.2. Ochratoxin A

OTA (PubChem CID: 442530) [47] is the second most important mycotoxin from the public health perspective [39]. It is mainly nephrotoxic [57] and hepatotoxic [58]. Furthermore, it exhibits genotoxic, teratogenic, immunosuppressive, and neurotoxic effects [57,59] and they have been confirmed by Arenas-Huertero et al. [49] and by EFSA [60]. According to the IARC, OTA is classified in group 2B “*Possibly carcinogenic to humans*” [52].

OTA producers of *Aspergillus* species, especially *A. carbonarius*, *A. ochraceus*, *A. westerdijkiae*, *A steynii*, *A. lacticoffeatus*, *A. niger*, *A. sclerotioniger*, and *A. tubingensis*, are typical for areas with subtropical and tropical climate while producers of *Penicillium* species, such as mainly *P. verrucosum* and *P. nordicum*, are typical for areas with temperate or colder climate [59,61]. *Aspergillus melleus* and *A. alliaceus* are less typical OTA producers [62].

### 4.3. Citrinin

CIT (PubChem CID: 54680783) [47] is reported to have nephrotoxic, hepatotoxic, genotoxic, mutagenic, teratogenic, and cytotoxic effects [63,64]; they have been confirmed by EFSA [65]. According to IARC, CIT is classified in group 3 “*Not classifiable as to its carcinogenicity to humans*” [52].

CIT is produced primarily by *Penicillium citrinum* [61,66]. Other fungi from *Penicillium* species such as *P. expansum and P. verrucosum* are also able to produce CIT [61]. In addition, *Monascus purpureus* and *M. ruber* have also been confirmed to produce CIT [61,67].

### 4.4. Fumonisins

FMNs, of which fumonisin B_1_ (FB_1_) (PubChem CID: 2733487) and fumonisin B_2_ (FB_2_) (PubChem CID: 2733489) [47] are discussed in this review, are reported to have nephrotoxic, hepatotoxic, cardiotoxic, immunosuppressive, neurotoxic, teratogenic, embryotoxic, pulmotoxic, and cytotoxic effects [49,68,69]. According to IARC, FMNs are classified in group 2B “*Possibly carcinogenic to humans*” [52].

FMNs are primarily produced by *Fusarium* species, mainly represented by *F. verticillioides* [70,71] and *F. proliferatum* [54]. Furthermore, *Aspergillus niger* has been reported to produce FB_2_ [72,73].

### 4.5. Trichothecenes

TCT involved in this review (DON (PubChem CID: 40024), NIV (PubChem CID: 5284433), T-2 toxin (T-2) (PubChem CID: 5284461) and HT-2 toxin (HT-2) (PubChem CID: 10093830) [47]) are reported to have genotoxic, mutagenic, teratogenic, immunosuppressive, hepatotoxic, neurotoxic, and hematoxic effects [68,74,75,76]. According to IARC, TCT (DON, NIV, T-2) are classified in group 3 “*Not classifiable as to its carcinogenicity to humans*” [52].

In food, TCT are produced primarily by *Fusarium* species [70,77,78], such as *F. graminearum* [54], *F. culmorum*, *F. cerealis* [70,78], and *F. crookwellense* in case of DON or NIV, and *F. poae, F. equiseti* and *F. acuminatum* in case of T-2 and its metabolite HT-2 [70,77].

### 4.6. Zearalenone

ZEA (PubChem CID: 5933650) [47] has been reported to have estrogenic, genotoxic, mutagenic, teratogenic, immuno-suppressive, and hematoxic effects [68,79]. According to IARC, ZEA is classified in group 3 “*Not classifiable as to its carcinogenicity to humans*” [52]. In food, ZEA is produced by *Fusarium* species represented by *F. graminearum, F. culmorum*, and *F. crookwellense* [70,77,80].

### 4.7. Alternaria Mycotoxins

*Alternaria* mycotoxins, such as alternariol (AOH) (PubChem CID: 5359485), altenuene (ALT) (PubChem CID: 5359485), or tenuazonic acid (TEA) (PubChem CID: 54683011) [47], have been reported to be genotoxic, mutagenic, teratogenic, and cytotoxic [49,81]. As for IARC classification, none of the *Alternaria* mycotoxins is listed, although supposed esophageal carcinogenic effects were reported [82].

*Alternaria* mycotoxins are produced by the *Alternaria* species [81]. Those that contaminate foods include *A. alternata* [83,84], *A. tenuissima, A. arborescent* [85], *A. tangelonis*, and *A. turkisafria* [86].

### 4.8. Sterigmatocystin

Sterigmatocystin (STEG) (PubChem CID: 5280389) [47] has been reported to possess hepatotoxic, nephrotoxic, genotoxic, mutagenic, and teratogenic effects [87]. According to IARC, STEG is classified in group 2B “*Possibly carcinogenic to humans”* [52] because it can induce tumors including hepatocellular carcinomas, liver haemangiosarcomas, angiosarcomas in brown fat, and lung adenomas in several species [87]. However, in comparison with AFB_1_, STEG toxicity has been assessed to be 10 or even up to 100 times lower [88,89]. Due to the minor significance of STEG in this review, its chemical structure is not shown in Table 2.

STEG is produced by more than 50 fungal species [87]. *Aspergillus versicolor* and *Emericella nidulans* (*anamorph: A. nidulans*) [62] are the main producers in food commodities as they can produce STEG in high amounts, compared to *A. flavus* and *A. parasiticus* which convert a part of STEG into O-methylsterigmatocystin a direct precursor of AFB_1_, resulting in lower STEG production [90,91].

## 5. International Regulation of Aflatoxins and Ochratoxin A in Spices

On the global level, the debate on fixing the limits on mycotoxins in spices seems to be relatively recent. In 2015, the Codex Alimentarius Commission or, more precisely, its Committee on Contaminants in Foods (CCCF) agreed to start working on a Code of practice for the prevention and reduction of mycotoxin contamination in spices and combinations of spices [92]. In the same year, the feasibility of establishing the maximum levels for selected spices was also discussed as a separate topic in the CCCF. It was India which, in 2014, initiated the discussion (the 8th Session; March 2014) and which simultaneously proposed to establish the maximum levels with respect to (i) total AFs, (ii) AFB_1_, and (iii) OTA in five different spices occupying a prominent place in the global trade with spices, namely, in dried or dehydrated forms of nutmeg, chili/paprika, ginger, pepper, and turmeric [92]. Upon the Indian proposal, the electronic working group was set up to deal with the issue. Reaching a consensus on establishing the maximum limits for total AFs and OTA in the proposed spices, however, turned out to be a complex process. While some states argued that more conclusive data on the occurrence of mycotoxins in spices were needed, others opined that the general level of the consumption of spices was too low to justify establishing the maximum limits for mycotoxins contained in spices. Due to the diverging views of different states, in 2018, the CCCF decided to temporarily discontinue works on establishing the maximum limits and give time to member states to implement the Code of Practice for the prevention and reduction of mycotoxins in spices adopted in 2017 [93]. Upon the implementation of the Code of Practice for the prevention and reduction of mycotoxins in spices, in a three-year horizon, the new data on the occurrence of mycotoxins in spices should be obtained, and based on them, the issue of establishing their maximum limits in spices should be re-examined by the CCCF. Nevertheless, the levels of 20/30 μg/kg for total AFs and the level of 20 μg/kg for OTA have been retained as the points of departure for future discussion [93].

Thus, for the time being, the most extensive regulation of the presence of mycotoxins in spices on an international level can be found in EU law. On the grounds of powers conferred by Article 2(3) of Council Regulation (EEC) No 315/93 [94], the European Commission adopted Commission Regulation (EC) No 1881/2006 of 19 December 2006 setting maximum levels for certain contaminants in foodstuffs [15].

Section 2 of the Annex to the Regulation No 1881/2006 fixes the maximum levels of selected mycotoxins in different foodstuffs including spices and licorice. Section 2 primarily establishes the maximum levels of AFs in *Capsicum* spp. (including chilies, chili powder, cayenne, and paprika), peppers, nutmeg, ginger, turmeric, and in the mixtures of these spices; see Table 3. In addition, Section 2 lays down the maximum limits for OTA in the same spices, their mixtures, and also in licorice; see Table 4. The maximum limits for OTA in spices evolved in a rather complex way. They were first laid down in Regulation No 105/2010 [95], later, they were amended by Regulation No 594/2012 [96]. The present maximum levels have been established by Regulation No 2015/1137.

In other regions, the regulation of the presence of mycotoxins in spices mostly depends on the sole appreciation of individual states. In this respect, data gathered by the CCCF in a direct link with the discussion on establishing the maximum limits for mycotoxins in spices provide an interesting insight on the existing limits in the Codex member states [93]. 

As it is evident from these data, at present, a fair number of the Codex member states have fixed the maximum limits on AFs in spices. These limits range from 1 μg/kg (Honduras) to 30 μg/kg (India). 

In the case of OTA, the same data suggest the situation is different in that the number of states which have laid down the maximum limits for the presence of OTA in spices seems to be markedly lower. The lowest limit of 10 μg/kg has been reported from Armenia while the highest one of 30 μg/kg has been reported from Brazil. However, even a lower limit has applied in South Korea where, at least in some spices (such as red pepper), the maximum limit for the presence of OTA has been related to be 7 μg/kg [97]. 

When it comes to identifying spices in which the presence of AFs and OTA is regulated, the approaches differ. While many states have limits fixed for all the foodstuffs, in other states, there are specific limits for spices in general or only for specific spices (such as chili or nutmeg).

These observations can be exemplified by the regulatory practice of several states which play a prominent role in the global trade in spices.

In India, which has initiated the discussion on the regulation of mycotoxins in spices on the global level, the maximum limits are prescribed for AFs in spices by the Food Safety and Standards Authority [98]. Currently, the maximum limit is 30 μg/kg. However, no limits on OTA in spices have been reported.

In China, the presence of mycotoxins in foodstuffs is currently regulated by the National Food Safety Standard of Maximum Levels of Mycotoxin in Foods (GB 2761-2012) which is based on comparative analysis of international and national standards and came into force in October 2011 [99].

In 2017, the National Standard has been updated by the National Food Safety Standard for Maximum Levels of Mycotoxins in Foods (GB 2761-2017), and in January 2020, the public consultation on its revision was launched [100]. While under the National Standard, the maximum level is set at 5.0 μg/kg for AFB_1_ in spices, it does not seem that specific maximum limits would apply with regard to other mycotoxins in spices.

In Brazil, before 2011, only the presence of AFs in some selected commodities such as peanuts was subject to legal regulation. Under the impact of the introduction of regulatory limits on international and the EU level, in 2011, however, the Brazilian Surveillance Agency (ANVISA) established the limits for six mycotoxins, which were amended in 2017 [101]. The existing regulation now applies to more than 20 categories of foodstuffs including spices. As for AFs (B_1_, B_2_, G_1_, G_2_), the maximum limits are fixed at 20 μg/kg; as for OTA, the maximum limit equals 30 μg/kg.

In the USA, the world’s largest spice consumer, in case of AFs (B_1_, B_2_, G_1_, G_2_), the action levels for their presence in foodstuffs have been laid down by the Food and Drug Administration (FDA) since 1965. Since 1969, the action level has been set at 20 μg/kg for all foodstuffs intended for human consumption, except milk [102]. The action levels are understood as levels above which the foodstuffs will be considered to be adulterated, which means the FDA is allowed to bring regulatory and enforcement action under the Federal Food, Drug, and Cosmetic Act (FFDC Act). As far as OTA is concerned, the FDA has not been reported to have established any action or guidance levels.

## 6. Mycotoxins and Microfungi in Spices from the Perspective of Research in the Last Five Years (Since 2015)

This review summarizes the studies concerning mycotoxins and their producers in spices over the last five years—since 2015. For the evaluation of the positivity on microfungi or mycotoxins, the following six-level scale was established: (i) none (0%), (ii) rare (up to 5%), (iii) low (up to 25%), (iv) moderate (up to 50%), (v) high (up to 75%), and (vi) very high (more than 75%) occurrence of positive results. This scale was used for the evaluation of the percentage of studies with a positive incidence of microfungi/mycotoxins in a given spice (a study with at least one positive sample, hereinafter referred to as “positive study”) in the total number of publications dealing with related microfungi/mycotoxins in spice. The same scale was used in case of the percentage of samples with a positive finding on mycotoxins in the total number of samples throughout all publications involved. However, it is important to consider the number of baseline studies, because the listed percentages are the more conclusive, the more studies they are based on.

### 6.1. Mycotoxins in Spices Overview

A total of 48 studies altogether covering 17 mycotoxins in 38 spices were included. Namely, these publications cover (the numbers in brackets indicate the number of publications related to the kind of spice or type of mycotoxin) allspice (*Pigmenta officinalis*) (2), anise (*Pimpinella anisum*) (5), basil (*Ocimum basilicum*) (5), bay leaf (*Laurus nobilis*) (6), caraway (*Carum carvi*) (7), cardamom (*Elateria cardamomum*) (7), carom (*Trachyspermum ammi*) (1), chili (*Capsicum* spp.) (30), cinnamon (*Cinnamomum burmannii*) (11), cloves (*Eugenia caryophyllata*) (8), coriander (*Coriandrum sativum*) (10), cumin (*Cuminum cyminum*) (9), cumin black (*Nigella sativa*) (3), curry (3), dawdawa (*Parkia biglobosa*) (2), fennel (*Foeniculum vulgare*) (12), fenugreek (*Trigonella foenum-graecum*) (4), garlic (*Allium sativum*) (5), ginger (*Zingiber officinale*) (14), licorice (*Glycyrrhiza glabra*) (3), mace (*Myristica fragrans*) (1), marjoram (*Majorana hortensis*) (3), mint (*Mentha piperita*) (5), mustard (*Sinapis* spp.) (3), nutmeg (*Myristica fragrans*) (12), onion (*Allium* spp.) (3), oregano (*Origanum vulgare*) (5), paprika (*Capsicum* spp.) (6), parsley (*Allium schoenoprasum*) (3), pepper black (*Piper nigrum*) (23), pepper white (*Piper nigrum*) (6), rosemary (*Salvia rosmarinus*) (6), saffron (*Crocus* spp.) (2), sage (*Salvia* spp.) (4), star anise (*Illicium verum*) (1), sumac (*Rhus coriaria*) (3), thyme (*Thymus vulgaris*) (10), and turmeric (*Curcuma longa*) (11) in which the following mycotoxins were analyzed: AFB_1_ (33), AFB_2_ (19), AFG_1_ (19), AFG_2_ (18), OTA (20), CIT (4), ZEA (5), FB1 (9), FB2 (7), DON (4), NIV (3), T-2 (5), HT-2 (4), ALT (2), AOH (3), TEA (2), and STEG (4). 

The percentage of positive studies of the total number of studies dealing with related mycotoxin and spice are shown in Table S1 of the Supplementary Materials (for mycotoxins produced by *Aspergillus* and *Penicillium* genera), Table S2 of the Supplementary Materials (for *Fusarium* mycotoxins) and Table S3 of the Supplementary Materials (for *Alternaria* mycotoxins). Similarly, the percentage of the total sum of positive samples to the total sum of tested samples for each unique spice and mycotoxin combination throughout all included publications are shown in Table 5, Table 6 and Table 7.

#### 6.1.1. Aflatoxins

As can be seen above, AFs (mainly AFB_1_) are undoubtedly the most frequently analyzed mycotoxins in spices. In terms of AFs, studies are most often concerned with chili, black pepper, ginger, fennel, turmeric, coriander, cinnamon, nutmeg, and thyme, in descending order. The occurrence of total AFs in the above-mentioned spices is usually high to very high. In the following summaries of positive findings, only aflatoxin occurrence supported by at least 5 studies or at least 30 samples will be described in more detail.

*Aflatoxin B*_1._ Number of AFB_1_-positive studies has been proven as very high in ginger, chili, and turmeric; as high in black pepper, cumin, coriander, and cinnamon; and as moderate in fennel, caraway, thyme, and nutmeg—see Table S1 of the Supplementary Materials.

The AFB_1_ occurrence has been proven as high in ginger, chili, fenugreek, turmeric, and coriander; as moderate in paprika, cumin, black pepper, nutmeg, and fennel; as low in caraway, cinnamon, and white pepper; as rare in licorice and thyme, and none in oregano and basil—see Table 5.

The highest AFB_1_ concentrations in different spices have been reported in nutmeg (1632.2 µg/kg) in Indonesia [105], chili (156.0 µg/kg) in Nigeria [106], paprika (155.7 µg/kg) in Italy [107], black pepper (75.8 µg/kg) in Pakistan [108], licorice (57.0 µg/kg) in Egypt, black cumin (56.8 µg/kg) in Egypt [109], ginger (39.8 µg/kg) in Iran [110], parsley (27.4 µg/kg) in Egypt [109], saffron (26.5 µg/kg) in Algeria [111], fennel (21.7 µg/kg) in Malaysia [112], mustard (18.2 µg/kg) and thyme (16.8 µg/kg) in Egypt [109], and coriander (11.0 µg/kg) in Malaysia [112].

Aflatoxin B_2._ Several AFB_2_-positive studies have been proven as high in chili, turmeric, ginger, and black pepper; as moderate in coriander and fennel; and as low in cinnamon—see Table S1 of the Supplementary Materials.

The AFB_2_ occurrence has been proven as moderate in ginger; as low in turmeric, chili, caraway, paprika, coriander, fenugreek, black pepper, nutmeg, fennel, and cumin; as rare in white pepper; and as none in cinnamon and licorice—see Table 5.

The highest AFB_2_ concentrations in different spices have been reported in chili (33.3 µg/kg) in Indonesia [113], paprika (9.9 µg/kg) in Italy [107], parsley (2.5 µg/kg) in Egypt [109], and fennel (2.3 µg/kg), turmeric (1.7 µg/kg) and coriander (1.6 µg/kg) in Malaysia [112].

Aflatoxin G_1._ Number of AFG_1_-positive studies has been proven as high in turmeric and cumin and as moderate in chili, black pepper, fennel, cinnamon, and ginger—see Table S1 of the Supplementary Materials.

The AFG_1_ occurrence has been proven as moderate in fennel and white pepper; as low in cumin, turmeric, paprika, fenugreek, cinnamon, ginger, chili, coriander, and black pepper; and as rare in nutmeg, caraway, and licorice—see Table 5.

The highest AFG_1_ concentrations in different spices have been reported in paprika (318.1 µg/kg) in Italy [107], anise (157.5 µg/kg), thyme (41.2 µg/kg), black pepper (31.5 µg/kg), rosemary (12.9 µg/kg), mustard (10.5 µg/kg) and parsley (8.1 µg/kg) in Egypt [109], and chili (7.0 µg/kg) in Malaysia [114].

Aflatoxin G_2._ Number of AFG_2_-positive studies has been proven as moderate in chili, cumin, ginger, coriander, black pepper, and fennel and as rare in cinnamon and turmeric—see Table S1 of the Supplementary Materials.

The AFG_2_ occurrence has been proven as moderate in white pepper; as low in fenugreek, turmeric, coriander, paprika, black pepper, and chili; as rare in fennel, cumin, ginger, and caraway; and none in nutmeg, cinnamon, and licorice—see Table 5.

The highest AFG_2_ concentrations in different spices have been reported in paprika (45.4 µg/kg) in Italy [107], black pepper (16.0 µg/kg) in Egypt, mustard (7.6 µg/kg) in Egypt [109], chili (1.5 µg/kg) in Turkey [115], and cinnamon (0.4 µg/kg) in Iran [116].

#### 6.1.2. Ochratoxin A

OTA is the second most frequently analyzed mycotoxin in spices, after AFs. In terms of OTA, studies are most often concerned with black pepper, chili, ginger, fennel, and turmeric, in descending order, where its occurrence is high to very high. In the following summaries of positive findings, only OTA occurrence supported by at least 5 studies or at least 30 samples will be described in more detail.

The number of OTA-positive studies has been proven as very high in turmeric, chili, and ginger and as high in black pepper and fennel—see Table S1 of the Supplementary Materials.

The OTA occurrence has been proven as high in paprika and mace; as moderate in turmeric, ginger, fenugreek, cardamom, chili, black pepper, caraway, licorice, coriander, and fennel; as low in white pepper, cinnamon, and cumin; and none in oregano, clove, thyme, and basil—see Table 5.

The highest OTA concentrations in different spices have been reported in chili (907.5 µg/kg) in Ivory Coast [117], paprika (177.4 µg/kg) in Italy [107], black pepper (79.0 µg/kg) in Sri Lanka [118], cardamom (78.0 µg/kg) in Saudi Arabia [119], nutmeg (60.7 µg/kg) and licorice (36.7 µg/kg) in the Czech Republic [120], cumin (20.4 µg/kg) in Malaysia [112], cinnamon (16.1 µg/kg) in Iran [121], ginger (12.7 µg/kg) in the Czech Republic [120], curry (9.6 µg/kg) in Malaysia [112], turmeric (8.5 µg/kg) in Iran [121], garlic (5.1 µg/kg) in Lebanon [9], and white pepper (4.9 µg/kg) in Cameroon [35].

#### 6.1.3. Citrinin

Very few studies deal with CIT in spices—only 1 to 3 studies pertain to a single spice at a time. Publications mentioning CIT-positive findings deal with black pepper, chili, coriander, cumin, fenugreek, ginger, and licorice. On the contrary, CIT has not been found in basil, caraway, fennel, nutmeg, oregano, thyme, and turmeric, although they have been tested—see Table S1 of the Supplementary Materials.

The CIT occurrence has been proven as moderate in chili, ginger, coriander, and fenugreek; as low in black pepper and licorice; and none in basil, nutmeg, oregano, thyme, and turmeric—see Table 5.

#### 6.1.4. Fumonisins

As in the case of CIT, there are not many studies for FMNs in spices—only 1-4 and 1-2 studies dealing with FB_1_ and FB_2_, respectively, pertain to a single spice at a time. Studies with positive findings of FMNs in spices are rather rare; however, some publications in connection with positive findings in black pepper, licorice, nutmeg, mint, and thyme for FB_1_; chili for FB_2_; and paprika, onion spice and dawadawa for both of them have been published. On the contrary, neither of FMNs have been found in many kinds of spices—see Table S2 of the Supplementary Materials.

The FB_1_ occurrence has been proven as moderate in paprika and licorice; as low in mint and garlic; as rare in thyme; and as none in black pepper, oregano, and basil—see Table 6.

The FB_2_ occurrence has been proven as high in paprika and as none in garlic and licorice.

The highest FB_1_ concentrations in different spices have been reported in onion (591.0 µg/kg) in South Africa [133], garlic (540.0 µg/kg) of unknown origin [142], mint (256.0 µg/kg) in Turkey [143], paprika (243.9 µg/kg) in Italy [107], dawadawa (165.0 µg/kg) in Nigeria [34], black pepper (135.0 µg/kg) from Sri Lanka [118], thyme (125.0 µg/kg) in Turkey [143], licorice (39.3 µg/kg) in China [141], and nutmeg (25.0 µg/kg) originated in Indonesia [123].

The highest FB_2_ concentrations in different spices have been reported in onion (4537.0 µg/kg) in South Africa, chili (425.0 µg/kg) in South Africa [133], paprika (176.9 µg/kg) in Italy [107], and dawadawa (170.0 µg/kg) in Nigeria [34].

#### 6.1.5. Trichothecenes (DON, NIV, T-2, HT-2)

As with CIT and FMNs, there are not many studies for TCT in spices, including DON, NIV, T-2, and HT-2—see Table S2 of the Supplementary Materials. None of the TCT has been detected in basil, nutmeg, black pepper, and oregano, while all of the above-mentioned toxins have been detected in paprika at low to moderate levels. For thyme, DON has been detected at a low level, while none of the other TCT has been detected—see Table 6.

The highest concentrations in different spices have been reported in paprika (59.8 µg/kg) in Italy [107] and licorice (11.0 µg/kg) in China [141] for DON, in paprika (243.9 µg/kg) in Italy [107] for NIV, in dawadawa (32.0 µg/kg) in Nigeria [34] and paprika (27.1 µg/kg) in Italy [107] for T-2, and in paprika (75.9 µg/kg) in Italy [107] and dawadawa (58.0 µg/kg) in Nigeria [34] for HT-2.

#### 6.1.6. Zearalenone

ZEA is one of the least analyzed mycotoxins in this review. No more than one study pertains to a single spice—see Table S2 of the Supplementary Materials. The ZEA occurrence has been proven as very high in paprika (up to 53.6 µg/kg) in Italy [107]; as moderate in dawadawa (up to 86.0 µg/kg) in Nigeria [34]; as low in thyme (up to 209.0 µg/kg) originated in Poland [123] and licorice (up to 8.8 µg/kg) in China [141]; and as none in chili originated in Korea [137], basil originated in India, nutmeg originated in Indonesia, oregano originated in Turkey, and black pepper originated in Brazil and Vietnam [123]—see Table 6.

#### 6.1.7. *Alternaria* Mycotoxins

*Alternaria* mycotoxins (ALT, AOH, TEA) are rarely studied in spices, as most data originated in just one publication [10]—see Table S3 of the Supplementary Materials. Moreover, very few samples per single spice have been tested.

All above mentioned *Alternaria* mycotoxins have been confirmed in cinnamon, ginger, chili and paprika, but no other findings have been found in anise, basil and parsley. In addition, among *Alternaria* mycotoxins, TEA has been found in most of spice samples: bay leaf, caraway, cardamom, cinnamon, cloves, coriander, cumin, fennel, fenugreek, garlic, ginger, chili, marjoram, mint, nutmeg, onion, oregano, paprika, black pepper, white pepper, rosemary, sage, sumac, thyme, and turmeric. More details about single *Alternaria* toxins and other spices are shown in Table 7.

The highest ALT concentrations in different spices have been reported in clove (11.7 µg/kg) in Lebanon [10]; paprika (40.3 µg/kg) in Italy [107]; and ginger (5.2 µg/kg), chili (3.6 µg/kg), and turmeric (2.8 µg/kg) in Lebanon [10]. The highest AOH concentrations in different spices have been reported in licorice (520.6 µg/kg) in China [141]; paprika (428.4 µg/kg) in Italy [107]; and white pepper (319.7 µg/kg), black pepper (89.0 µg/kg), garlic (57.4 µg/kg), oregano (13.5 µg/kg), nutmeg (12.7 µg/kg), mint (11.8 µg/kg), allspice (8.0 µg/kg), sumac (6.6 µg/kg), and ginger (5.4 µg/kg) in Lebanon [10]. The highest TEA concentrations in different spices have been reported in paprika (8248.5 µg/kg) in Italy [107] and rosemary (50.4 µg/kg), bay leaf (48.2 µg/kg), nutmeg (22.0 µg/kg), white pepper (20.3 µg/kg), and clove (14.9 µg/kg) in Lebanon [10].

#### 6.1.8. Sterigmatocystin

STEG has been found in oregano (unknown positivity, up to 28.0 µg/kg) originated in Turkey [123], at low level in paprika (14.3%, 1/7, 18.0 µg/kg) in South Africa [133], and at rare level in thyme (4%, 2/50, up to 14 µg/kg) originated in Poland [123]. Black pepper and chili have been found positive in Sri Lanka at moderate levels (43.9%, 36/82, 49.0 µg/kg and 38.4%, 33/86, up to 32 µg/kg, respectively) [118], while no STEG has been detected in 50 samples of black pepper originated in Brazil and Vietnam [123] and in 18 samples of chili in South Africa [133]. STEG has been detected in none of the following spices: 50 basil samples originated in India [123], 31 licorice samples from China [141], 50 nutmeg samples originated in Indonesia [123], or 8 onion samples from South Africa [133]. For very little data, STEG is further discussed neither in the text nor in the table.

### 6.2. Microfungi in Spices Overview

A total of 25 studies altogether covering 14 microfungi in 33 spices were included. These publications cover (the numbers in brackets indicate the number of publications related to the kind of spice or microfungi) anise (3), basil (1), bay leaf (2), caraway (6), cardamom (6), chili (14), cinnamon (8), cloves (8), coriander (6), cumin (5), cumin black (2), curry (4), fennel (8), fenugreek (3), garlic (3), ginger (7), licorice (1), mace (1), marjoram (1), mint (1), mustard (3), nutmeg (10), oregano (2), paprika (2), parsley (1), pepper black (12), pepper white (6), rosemary (3), saffron (3), star anise (1), sumac(2), thyme (3), and turmeric (5) in which the following microfungi were analyzed: *Aspergillus flavus* (20), *A. parasiticus* (13), *A. niger* (20), *A. carbonarius* (4), *A. tamarii* (8), *A. terreus* (6), *A. versicolor* (7), *A. ochraceus* (8), *Penicillium citrinum* (13), *P. verrucosum* (3), *Fusarium verticillioides* (3), *Alternaria alternata* (5), *Rhizopus nigricans* (3), and *R. oryzae* (4). 

The percentage of positive studies to the total number of studies concerning each unique spice and microfungi combination are shown in Table 8 (for *Aspergillus* spp., *Penicillium* spp., and *Fusarium* spp.), Table 9 (for *Aspergillus* species), and Table 10 (for *Penicillium, Fusarium, Alternaria*, and *Rhizopus* species). 

*Aspergillus, Penicillium*, and *Fusarium* genera are the most important mycotoxin producers in various commodities [39], which also applies to spices in which they are commonly present, as can be seen in Table 8. Out of the mentioned microfungi genera, spices are predominantly contaminated by *Aspergillus* followed by *Penicillium* and then by *Fusarium* strains. In the following summary, only microfungi occurrences supported by at least 5 individual studies are described in more detail.

#### 6.2.1. *Aspergillus* Species

*Aspergillus* species are in the vast majority of spices. The occurrence is very high in chili, fennel, ginger, caraway, coriander, white pepper, turmeric, black pepper, nutmeg, cardamom, and cumin and moderate in cinnamon and cloves. Based on all included studies, some *Aspergillus* strains were isolated from all spices involved in this review except for star anise, which was only analyzed once and with negative results—see Table 8. 

Of the *Aspergillus* species, *A. niger* is most common in spices, followed by *A. flavus* and *A. ochraceus*. The occurrence of *A. niger* is very high in black pepper, cardamom, chili, and fennel, high in cinnamon, ginger, and nutmeg, and low in cloves. The occurrence of *A. flavus* is very high in chili, black pepper, cardamom, and white pepper; high in nutmeg and fennel; and moderate in cloves and cinnamon. The occurrence of *A. ochraceus* is very high in black pepper and moderate in chili and fennel. As for the less significant species, the occurrence of *A. tamarii* is very high in chili and high in nutmeg, and the occurrence of *A. parasiticus* is high in black pepper, ginger, and chili; moderate in cloves; and low in fennel. The other *Aspergillus* species and data supported by less than five studies are shown in more detail in Table 9.

#### 6.2.2. *Penicillium* Species

Compared to *Aspergillus* spp., the occurrence of *Penicillium* spp. is slightly lower but overall, still significant, as it has been very high in caraway and turmeric; high in black pepper, chili, nutmeg, and coriander; moderate in cardamom, cinnamon, fennel, and ginger; and low in cloves.

Based on all included studies, *Penicillium* spp. was isolated from all spices included in the present review except for four cases: curry, garlic, star anise, and sumac—see Table 8.

The two *Penicillium* species considered in this review were *P. citrinum* and *P. verrucosum*, among which the second mentioned is studied rather rarely but in most cases came out positive. The occurrence of *P. citrinum* is moderate in nutmeg, black pepper, and chili; low in fennel; and none in cloves—see Table 10.

#### 6.2.3. *Fusarium* Species

*Fusarium* spp. occurs in spice substantially less than *Aspergillus* spp. or *Penicillium* spp., although its occurrence is still very high in chili, then high in fennel and turmeric, moderate in cardamom and black pepper, and low in caraway. Apart from oregano which has not been tested for *Fusarium* spp. and again considering all included studies, this genus has been confirmed in 16 out of 32 involved spices—see Table 8. *F. verticillioides* (= *F. moniliforme)* is very little studied with only 1-3 relevant studies per spice. It was confirmed to occur in at least one case in cardamom, fennel, fenugreek, ginger, chili, mace, black pepper, thyme, and turmeric—see Table 10.

#### 6.2.4. Other Microfungi (*Alternaria alternata*, *Rhizopus nigricans* and *Rhizopus oryzae*)

Only a few publications deal with *Alternaria alternata* and *Rhizopus nigricans* and even fewer with *R. oryzae* in spices; therefore, it is not possible to summarize them based on the previously established threshold of five studies. All three have been confirmed to appear in cumin and coriander. In addition, *A. alternata* has been found in anise, cardamom, fenugreek, chili, and mustard; *R. nigricans* in mace, mustard, and black pepper; and *R. oryzae* in bay leaf, cardamom, fennel, fenugreek, ginger, chili, mace, black pepper, sumac, and turmeric—see Table 10.

## 7. Mycotoxin Levels in Spices in Relation to European Legislation

The concentrations of AFs and/or OTA in spices often exceeded the maximum permissible limit (MPL) set by EU legislation in involved studies where MPL for AFs and AFB_1_ were exceeded more often than in case of OTA. Chili and paprika (*Capsicum* spp.) seem to be the most problematic spices. Aflatoxin concentrations exceeded MPL in 10 of 12 studies (83.3%) and 3 of 3 studies (100%) for total AFs and 13 of 18 studies (72.2%) and 2 of 3 studies (66.7%) for AFB_1_, respectively. In the case of OTA, MPL was exceeded by 50.0% for both chili (6/12) and paprika (2/4). Nutmeg seems to be also problematic, as its concentration exceeded MPL in 3 of 4 studies (75.0%) for total AFs and 2 of 3 studies (66.7%) for OTA. However, the concentration of AFB_1_ exceeded MPL only in one of 6 studies (16.7%). On the contrary, in the case of white pepper, MPL was exceeded in a single study dealing with total AFs (1/4, 25%) and was not exceeded in any of 3 studies concerning AFB_1_ and 4 studies concerning OTA—see Table 11.

## 8. Mycotoxins in Spices Based on RASFF

**Figure 2:**
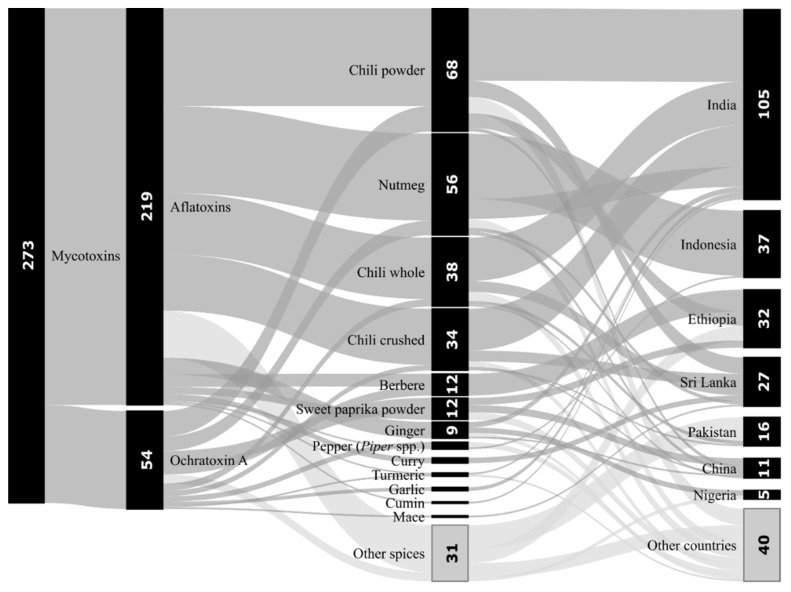
Notifications of aflatoxins and ochratoxin A in spices by the Rapid Alert System for Food and Feed (RASFF) in 2015–2019. Notes: Number of notifications in brackets: Pepper, *Piper* spp. (4), Curry (3), Turmeric (2), Garlic (2), Cumin (1), and Mace (1). The category “Other spices” includes fasika spice, kebab spice, suya pepper, and other various spice mixtures. The category “Other countries” includes all countries with less than 5 mycotoxin notifications for spices: Bangladesh, Croatia, France, Germany, Ghana, Grenada, Hong Kong, Italy, Kosovo, Kuwait, Lebanon, Malawi, Netherlands, Peru, Spain, Thailand, Turkey, United Kingdom, and Vietnam. Processed according to RASFF [156].

Based on Rapid Alert System for Food and Feed (RASFF) database from the last five years (2015-2019), in terms of several of mycotoxin notifications, the category “Herbs and spices” ranks third after categories “Nuts, nut products and seeds” and “Fruits and vegetables”. A total of 219 (80.2%) and 54 (19.8%) mycotoxin notifications relate to AFs and OTA in spices respectively, with 18 of the notifications concerning both. More than a half (51.3%) of the notifications include chilies (powdered, whole, and crushed), followed by nutmeg (20.5%). Each of the other spices, such as berbere spice, sweet powder, ginger, pepper, curry, and turmeric, represents less than 5% and cumin and mace even less than 1%. The most notifications originated in India (38.5%), far followed by Indonesia (13.6%), Ethiopia (11.7%), Sri Lanka (9.9%), Pakistan (5.9%), China (4.0%), and Nigeria (1.8%) and other countries—see Figure 2 [156]. Some of the highest values of aflatoxin contamination are shown in Table 12 [156].

## 9. Discussion

Mycotoxins in spices are quite often notified by the RASFF. Unfortunately, the RASFF data can be difficult to grasp due to occasional data inconsistency—e.g., inconsistent data format, missing unit, and inconsistent use of decimal point and comma (possibly leading to misunderstanding decimal for thousands separator). Data containing one of these ambiguities could not be included in overall data analysis due to possible distortion of the results—however, the amount of omitted data was not significant. Obviously, the frequency of notifications alone is not conclusive, as it is directly affected by the volume of production of the spice. Unfortunately, the worldwide production could not be carried out for each individual spice, since the FAOSTAT data of certain kinds of spices are grouped—e.g., in the group of four single spices “*Anise, badian, fennel, and coriander*” or the group of three single spices “*Nutmeg, mace, and cardamoms*”.

As evident from the studies included in this review, AFs and OTA are the most-commonly researched mycotoxins in spices, especially in chili and black pepper. However, for a better summary of all data, complete and accurate data (mainly concentration ranges, percentages of positive samples, and total numbers of analyzed samples) are needed, some of which are often lacking in many publications. Due to missing data, several publications in this summary had to be omitted.

In general, most spices appear to be prone to fungal infection and thus potentially mycotoxin contamination. Paprika, chili pepper, black pepper, white pepper, ginger, and turmeric seem to be one of the most critical in terms of mycotoxin contamination—often contaminated with all AFs and OTA. In addition, licorice is usually not contaminated with AFs, but quite often with OTA. All above-mentioned mycotoxins in spices are handled in EU legislation; however, many other spices are often contaminated not only with both AFs and OTA but even with other mycotoxins. None of those other spices and mycotoxins are handled in the EU legislation. Among others, e.g., AFs and OTA mainly in cardamom, mace, fenugreek, and other spices and CIT in chili, ginger, coriander, or fenugreek and *Fusarium* mycotoxins in paprika, onion, or chili pepper are all quite common; however, they are not addressed in the EU legislation.

Most original papers deal with spices that are already infamous for their mycotoxin contamination, namely, chilies and black pepper. Although these spices indeed appear to be the most crucial in terms of spice-related human mycotoxin exposure, and their analysis can obviously be expected to produce highly positive results, there are many other important spices. Of course, regional spices such as dawadawa can also be of a big concern in a given region and deserve no less attention than the major ones.

On the contrary, certain spices appear to be either resistant to fungal infection or possess the ability to inhibit mycotoxin production. In this review, these spices mainly include basil, cloves, mint, oregano, and thyme, which are only very rarely contaminated with any mycotoxins. Cases of uncontaminated spices remain in the background and are not discussed in the literature to a greater extent. Usually, these spices are only mentioned in relation to their essential oils, which can supposedly inhibit fungal activity and could possibly be the cause of those certain spices being contaminated rarely.

The essential oils of oregano, basil, and sage with their major compounds, thymol, methyl-cavicol, and thujone, respectively, supposedly inhibit *A. ochraceus* growth and its OTA production [157], so do essential oils of cinnamon, thyme, cloves, caraway, and anise [158,159]. On the contrary, oils of mint and oregano with major compounds menthol and linalool were reported to have no important inhibitory effect on the growth [157]. In this review, an inhibitory effect on the fungal growth can be partially confirmed in the case of coriander, cloves, and mint in which *A. ochraceus* was not detected in any of involved studies, while its presence was confirmed in case of oregano, basil, cinnamon, thyme, caraway, and anise. No data were available for confirming this effect for sage.

Similarly, essential oils of cinnamon and cloves (major compounds cinnamic aldehyde and eugenol, respectively [157]) and also thyme, mint, basil, caraway, and anise (thymol, menthon, methyl-chavicol, anithol) supposedly inhibit the growth of *A. parasiticus* [157,159]. In this review, the inhibitory effect can be supported in the case of caraway, mint, basil, and anise, where *A. parasiticus* was not detected. Its occurrence was relatively low in the case of cinnamon, which may be due to the inhibitory effect of the essential oil. However, in the case of both thyme and cloves, this inhibitory effect cannot be confirmed.

The inhibitory effect on the growth of *A. flavus* and *F. verticillioides* was reported in the case of essential oils of thyme, cinnamon, mint, basil, caraway, and anise [159]. In this review, the inhibitory effect can be supported only in the case of mint, basil, and anise for *A. flavus* and in the case of cinnamon, mint, basil, anise, and caraway for *F. verticillioides*, as the respective mold was not detected in the mentioned spices.

In addition, in case of cloves, thyme, and oregano, the occurrence of OTA and all AFs was mostly none or rare, although the occurrence of various fungi of *Aspergillus* and *Penicillium* genera has been confirmed, which may indicate the inhibitory effect on the mycotoxin production rather than the growth of the fungi.

## 10. Conclusions

Mycotoxins are considered potent pathogens. Some of them are highly carcinogenic. Food is the main source of mycotoxins in human body. Spices are a small but integral part of the diet of all people in the world. Therefore, spices are certainly not the main source of the supply of mycotoxins to the human body, but they can contribute to a considerable extent through continuous consumption. The control of mycotoxins in spices is a constantly evolving process, and the obtained data are very important not only for the realization of the dietary exposure to mycotoxins and health risk assessment but also for setting relevant legislation. AFs (mainly AFB_1_) and OTA are the most common mycotoxins in spices. However, compared to AFs and OTA, other mycotoxins have been insufficiently studied in spices, and thus, their share in the supply of mycotoxins is difficult to evaluate under the existing data. Among *Alternaria* mycotoxins, an honorable mention belongs to TEA due to its high incidence. Unfortunately, this fact is supported by very few studies. Even less data have been available in the case of CIT and *Fusarium* mycotoxins. As for microfungi, the most common species isolated from spices belong to *Aspergillus* and *Penicillium* and less to *Fusarium* genera. *A. niger* and *A. flavus* are considered to be dominant species isolated from the spice, followed by *A. ochraceus* and *P. citrinum*.

Based on the EU RASFF data, chili, nutmeg, and paprika powder have been the most problematic spices in terms of the frequency of exceeding maximum EU limits. Data from original papers, on which this review has been based, only confirm this conclusion. However, AFs, OTA, and also other mycotoxins have been proven to be present in relatively high amounts in many other spices as well. 

This review documents and emphasizes the importance of further monitoring of mycotoxins in common as well as less common spices. As proven, many spices are neglected in terms of mycotoxin monitoring but also have the potential for high contamination. Similarly, many mycotoxins are insufficiently monitored in spices, although their presence has been proven in this review. Given the findings from the included studies, it seems that the current legislation is rather incomplete, and inclusion of both less-common spices and less-common mycotoxins should be considered. It is therefore justified to advise authors to provide complete statistical data or full datasets in their studies, which may potentially be useful for setting new limits. Moreover, there is a need for regulation to be harmonized from country to country, depending on local dietary habits and needs. Nevertheless, more studies are needed to fix these maximum limits. Last but not least, it is also urgent to increase consumer awareness of the risk posed by mycotoxins in spices and their potential health impact.

## Figures and Tables

**Table 1:** Top 20 spice producers in the world in the last available year 2018.

Country	Category of Spice	Production (Tonnes)	Country	Category of Spice	Production (Tonnes)
India	1, 2, 5, 7, 8, 11	5,393,231	Pakistan	2, 5, 11	225,682
China	1, 2, 3, 4, 5, 6, 8, 9, 10, 11	1,163,542	Mexico	1, 2, 5, 6, 8, 9, 10, 11	206,232
Indonesia	3, 4, 5, 7, 8, 10, 11	651,075	Myanmar	2, 6, 11	186,190
Nepal	2, 5, 6, 7, 11	550,070	Canada	1, 6	186,052
Nigeria	2, 5, 11	446,793	Morocco	1, 2, 9, 11	157,365
Thailand	2, 5, 8, 11	419,348	Russian Fed.	1, 6	133,653
Vietnam	1, 2, 3, 8	397,770	Côte d’Ivoire	2, 5, 8, 11	125,097
Bangladesh	2, 5, 11	393,694	Ghana	2, 5, 8	119,388
Ethiopia	1, 2, 5, 6, 7, 8, 11	356,239	Brazil	8	101,274
Turkey	1, 2, 10, 11	299,487	Sri Lanka	3, 4, 5, 6, 7, 8, 11	100,745

Notes: Number of spice category: (1) Anise, badian, fennel, coriander; (2) Chilies and peppers, dry; (3) Cinnamon; (4) Cloves; (5) Ginger; (6) Mustard seed; (7) Nutmeg, mace, cardamoms; (8) Pepper, *Piper* spp.; (9) Peppermint; (10) Vanilla; (11) Spice not elsewhere specified. Processed according to FAOSTAT [37].

**Table 2:** Chemical characterization of mycotoxins.

Mycotoxin	Chemical Structure	Mycotoxin	Chemical Structure
AFB_1_	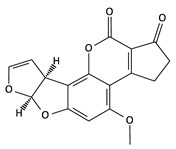	AFB_2_	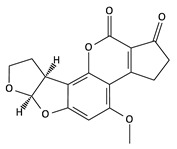
AFG_1_	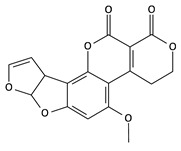	AFG_2_	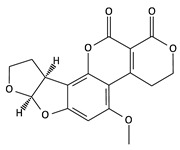
OTA	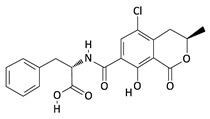	CIT	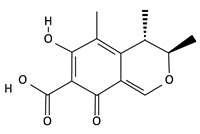
FB_1_	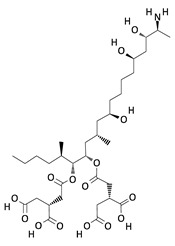	FB_2_	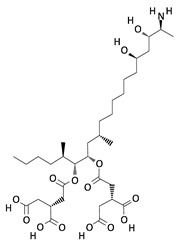
DON	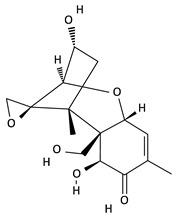	NIV	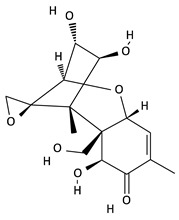
T-2	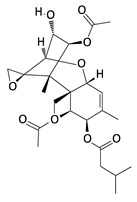	HT-2	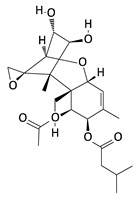
ZEA	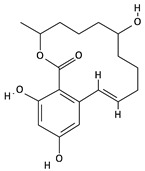	TEA	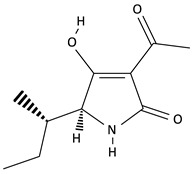
AOH	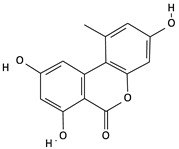	ALT	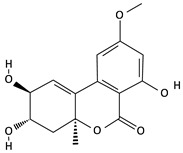

Note: AF—Aflatoxin B_1_, B_2_, G_1_, G_2_; OTA—Ochratoxin A; CIT—Citrinin; F—Fumonisin B_1_, B_2_; DON—Deoxynivalenol; NIV—Nivalenol; T-2—T-2 toxin; HT-2—HT-2 toxin; ZEA—Zearalenone; TEA—Tenuazonic acid; AOH—Alternariol; ALT—Altenuene. Processed according to PubChem [47].

**Table 3:** Maximum levels of aflatoxins in spices under EU legislation (Regulation No 1881/2006, as in force).

Foodstuff	AFB_1_ (µg/kg)	Total AFs ^a^ (µg/kg)	Reference
*Capsicum* spp. (dried fruits thereof, whole or ground, including chilies, chili powder, cayenne, and paprika) *Piper* spp. (fruits thereof, including white and black pepper) *Myristica fragrans* (nutmeg) *Zingiber officinale* (ginger) *Curcuma longa* (turmeric)	5	10	[15]
Mixtures of spices containing one or more of the abovementioned spices	5	10	[103]

Note: ^a^ AFs = Sum of aflatoxins B_1_, B_2_, G_1_ and G_2._

**Table 4:** Maximum levels of ochratoxin A in spices and licorice under EU legislation (Regulation No 1881/2006, as in force).

Foodstuff	OTA (µg/kg)	Reference
*Piper* spp. (fruits thereof, including white and black pepper) *Myristica fragrans* (nutmeg) *Zingiber officinale* (ginger) *Curcuma longa* (turmeric)	15	[96]
*Capsicum* spp. (dried fruits thereof, whole or ground, including chilies, chili powder, cayenne, and paprika)	20	[104]
Mixtures of spices containing one of the abovementioned spices	15	[96]
Licorice (*Glycyrrhiza glabra, Glycyrrhiza inflate* and other species)		[95]
Licorice root, ingredient for herbal infusion	20	
Licorice extract, for use in food in particular beverages and confectionary	80	

**Table 5:** Samples positivity: Natural occurrence of mycotoxins produced by *Aspergillus* and *Penicillium* species in spices in the last 5 years (since 2015).

Mycotoxin ^a^/Spice	AFB_1_			AFB_2_			AFG_1_			AFG_2_			AFs			OTA			CIT			Reference
	Positive ^b^ (%)		n ^c^	Positive (%)		n	Positive (%)		n	Positive (%)		n	Positive (%)		n	Positive (%)		n	Positive (%)		n	
Allspice	-	-	0	-	-	0	-	-	0	-	-	0	⏺	66.7	3	×	0.0	3	-	-	0	[9]
Anise	⏺	80.0	5	×	0.0	1	⏺	100	1	×	0.0	1	○	25.0	8	⏺	33.3	3	-	-	0	[9,109,111,122]
Basil	×	0.0	56	-	-	0	-	-	0	-	-	0	×	0.0	2	×	0.0	52	×	0.0	50	[9,110,123]
Bay leaf	×	0.0	25	×	0.0	18	○	11.1	18	○	22.2	18	×	0.0	6	×	0.0	2	-	-	0	[9,110,122,124]
Caraway	○	25.0	56	○	24.1	54	☆	3.7	54	☆	1.9	54	⏺	39.3	56	⏺	35.9	39	×	0.0	25	[8,9,111,120,124,125]
Cardamom	×	0.0	2	×	0.0	1	×	0.0	1	×	0.0	1	⏺	63.9	122	⏺	42.2	116	-	-	0	[9,109,119,122,126,127]
Carom	⏺	50.0	20	×	0.0	20	×	0.0	20	×	0.0	20	⏺	50.0	20	-	-	0	-	-	0	[125]
Chili	⏺	61.2	957	○	24.3	267	○	10.9	311	○	5.1	293	⏺	58.9	638	⏺	41.6	586	⏺	47.3	55	[8,9,106,108,110,111,112,113,114,115,116,117,118,120,121,122,128,129,130,131,132,133,134,135,136,137,138]
Cinnamon	○	17.6	51	×	0.0	39	○	15.4	39	×	0.0	39	⏺	32.3	62	○	20.5	39	-	-	0	[9,110,111,112,116,121,122,125,127,131]
Cloves	×	0.0	13	×	0.0	12	×	0.0	12	×	0.0	12	○	11.1	18	×	0.0	54	-	-	0	[9,122,127,131]
Coriander	⏺	56.5	46	○	19.0	42	○	8.1	62	○	6.5	62	⏺	53.1	64	⏺	31.1	45	⏺	40.0	30	[8,9,109,111,112,120,124,125]
Cumin	⏺	33.3	69	○	8.8	57	○	24.6	57	☆	3.5	57	⏺	56.5	62	○	5.7	35	○	21.4	28	[8,9,109,110,111,112,122,125]
Cumin, black	○	14.3	7	☆	4.8	21	⏺	100	1	☆	4.8	21	⏺	81.0	21	-	-	0	-	-	0	[109,110,125]
Curry	⏺	84.6	13	⏺	61.5	13	○	23.1	13	○	7.7	13	⏺	84.6	13	⏺	100	8	-	-	0	[112]
Dawadawa	⏺	100	12	-	-	0	-	-	0	-	-	0	-	-	0	○	16.7	12	-	-	0	[130]
Fennel	⏺	25.3	91	○	9.2	76	⏺	30.3	76	☆	3.9	76	⏺	54.0	113	⏺	29.1	79	×	0.0	25	[8,9,109,110,111,112,124,125,126,127,131]
Fenugreek	⏺	58.3	36	○	16.7	36	○	16.7	36	○	13.9	36	⏺	62.5	40	⏺	46.2	39	⏺	37.1	35	[8,9,109]
Garlic	-	-	0	-	-	0	-	-	0	-	-	0	×	0.0	2	⏺	50.0	2	-	-	0	[9]
Ginger	⏺	63.1	217	⏺	29.7	192	○	13.0	192	☆	2.6	192	⏺	59.4	165	⏺	47.9	213	⏺	44.4	36	[8,9,109,110,111,117,120,122,130,139,140]
Licorice	☆	3.1	32	×	0.0	32	☆	3.1	32	×	0.0	32	☆	3.1	32	⏺	32.6	43	○	6.5	31	[109,120,141]
Mace	-	-	0	-	-	0	-	-	0	-	-	0	⏺	63.3	30	⏺	60.0	30	-	-	0	[126]
Marjoram	⏺	100	1	×	0.0	1	×	0.0	1	×	0.0	1	⏺	33.3	3	⏺	50.0	2	-	-	0	[9,109]
Mint	×	0.0	25	×	0.0	16	×	0.0	16	×	0.0	16	×	0.0	19	×	0.0	3	-	-	0	[9,110,124]
Mustard	⏺	50.0	2	×	0.0	1	⏺	100	1	⏺	100	1	⏺	100	1	○	25.0	12	-	-	0	[109,120,127]
Nutmeg	⏺	27.9	104	○	13.2	53	☆	3.8	53	×	0.0	53	⏺	55.7	131	⏺	92.9	14	×	0.0	50	[9,105,109,120,123,127,135]
Onion	×	0.0	8	-	-	0	×	0.0	8	-	-	0	×	0.0	12	×	0.0	12	-	-	0	[9,133]
Oregano	×	0.0	79	×	0.0	29	×	0.0	29	×	0.0	29	☆	3.1	32	×	0.0	65	×	0.0	50	[9,123,124,131]
Paprika	⏺	47.6	42	○	22.6	31	○	18.4	38	○	6.5	31	⏺	43.9	41	⏺	60.4	53	-	-	0	[9,107,111,120,133]
Parsley	⏺	100	1	⏺	100	1	⏺	100	1	×	0.0	1	⏺	50.0	2	×	0.0	1	-	-	0	[9,109]
Pepper, black	⏺	31.0	226	○	13.8	80	○	7.5	120	○	5.7	140	⏺	44.8	203	⏺	36.0	264	○	20.7	92	[8,9,35,108,109,110,111,112,116,117,120,121,122,123,125,126,127,129,130,131,134]
Pepper, white	○	5.3	38	☆	2.6	38	⏺	26.3	38	⏺	26.3	38	⏺	55.0	40	○	21.1	38	-	-	0	[9,35,112,125,131]
Rosemary	○	14.8	27	⏺	29.6	27	☆	3.7	27	⏺	33.3	27	⏺	27.8	18	○	5.9	17	-	-	0	[9,109,124,131]
Saffron	⏺	50.0	4	-	-	0	-	-	0	-	-	0	×	0.0	1	×	0.0	1	-	-	0	[9,111]
Sage	⏺	33.3	3	×	0.0	1	⏺	100	1	×	0.0	1	⏺	50.0	4	⏺	33.3	3	-	-	0	[9,109,110]
Star anise	×	0.0	1	-	-	0	-	-	0	-	-	0	-	-	0	-	-	0	-	-	0	[127]
Sumac	×	0.0	9	-	-	0	-	-	0	-	-	0	×	0.0	2	×	0.0	2	-	-	0	[9,110]
Thyme	☆	2.7	73	×	0.0	13	○	7.7	13	×	0.0	13	○	6.3	16	×	0.0	53	×	0.0	50	[9,109,110,123,124]
Turmeric	⏺	57.1	70	○	24.6	65	○	24.6	65	○	8.2	85	⏺	51.9	129	⏺	49.0	104	×	0.0	35	[8,9,109,110,112,116,121,122,125,126]

Notes: ^a^ AFB_1_ = Aflatoxin B_1_, AFB_2_ = Aflatoxin B_2_, AFG_1_ = Aflatoxin G_1_, AFG_2_ = Aflatoxin G_2_, AFs = Aflatoxins, OTA = Ochratoxin A, CIT = Citrinin; ^b^ Positive = the percentage of positive samples; ^c^ n = the total number of samples related to mycotoxin and spice from all publications involved; × = none occurrence (0%), ☆ = rare occurrence (up to 5%), ○ = low occurrence (up to 25%), ⏺  = moderate occurrence (up to 50%), ⏺ = high occurrence (up to 75%), ⏺ = very high occurrence (more than 75%).

**Table 6:** Samples positivity: Natural occurrence of *Fusarium* mycotoxins in spices in the last 5 years (since 2015).

Mycotoxin ^a^/Spice	FB_1_			FB_2_			DON			NIV			T-2			HT-2			ZEA			Reference
	Positive^b^ (%)		n ^c^	Positive (%)		n	Positive (%)		n	Positive (%)		n	Positive (%)		n	Positive (%)		n	Positive (%)		n	
Basil	×	0.0	55	×	0.0	5	×	0.0	50	×	0.0	50	×	0.0	50	×	0.0	50	×	0.0	50	[123,143]
Bay leaf	×	0.0	19	×	0.0	1	-	-	0	-	-	0	×	0.0	18	×	0.0	18	-	-	0	[124,143]
Caraway	×	0.0	9	-	-	0	-	-	0	-	-	0	×	0.0	9	×	0.0	9	-	-	0	[124]
Chili	×	0.0	18	○	5.6	18	-	-	0	-	-	0	-	-	0	-	-	0	×	0.0	56	[133,137]
Coriander	×	0.0	17	×	0.0	8	-	-	0	-	-	0	⏺	33.3	9	×	0.0	9	-	-	0	[124,143]
Dawadawa	⏺	47.1	17	⏺	58.8	17	×	0.0	17	×	0.0	17	⏺	35.3	17	○	5.9	17	⏺	35.3	17	[34]
Fennel	×	0.0	11	-	-	0	-	-	0	-	-	0	×	0.0	11	×	0.0	11	-	-	0	[124]
Garlic	○	5.4	56	×	0.0	56	-	-	0	-	-	0	-	-	0	-	-	0	-	-	0	[142]
Licorice	⏺	38.7	31	×	0.0	31	☆	3.2	31	-	-	0	×	0.0	31	-	-	0	○	12.9	31	[141]
Mint	○	6.5	31	×	0.0	15	-	-	0	-	-	0	○	18.8	16	×	0.0	16	-	-	0	[124,143]
Nutmeg	-	-	0	-	-	0	×	0.0	50	×	0.0	50	×	0.0	50	×	0.0	50	×	0.0	50	[123]
Onion	⏺	37.5	8	⏺	87.5	8	-	-	0	-	-	0	-	-	0	-	-	0	-	-	0	[133]
Oregano	×	0.0	67	-	-	0	×	0.0	50	×	0.0	50	×	0.0	67	×	0.0	67	×	0.0	50	[123,124]
Paprika	⏺	50.0	38	⏺	73.7	38	⏺	38.7	31	⏺	48.4	31	○	19.4	31	○	19.4	31	⏺	87.1	31	[107,133]
Pepper, black	×	0.0	50	-	-	0	×	0.0	50	×	0.0	50	×	0.0	50	×	0.0	50	×	0.0	50	[123]
Rosemary	×	0.0	11	-	-	0	-	-	0	-	-	0	×	0.0	11	×	0.0	11	-	-	0	[124]
Thyme	☆	1.3	76	×	0.0	14	○	14.0	50	×	0.0	50	×	0.0	62	×	0.0	62	○	18.0	50	[123,124,143]

Notes: ^a^ FB_1_ = Fumonisin B_1_, FB_2_ = Fumonisin B_2_, DON = Deoxynivalenol, NIV = Nivalenol, T-2 = T-2 toxin, HT-2 = HT-2 toxin, ZEA = Zearalenone; ^b^ Positive = the percentage of positive samples; ^c^ n = the total number of samples related to mycotoxin and spice from all publications involved; × = none occurrence (0%), ☆ = rare occurrence (up to 5%), ○ = low occurrence (up to 25%), ⏺  = moderate occurrence (up to 50%), ⏺  = high occurrence (up to 75%), ⏺ = very high occurrence (more than 75%).

**Table 7:** Samples positivity: Natural occurrence of *Alternaria* mycotoxins in spices in the last 5 years (since 2015).

Mycotoxin ^a^/Spice	ALT			AOH			TEA			Reference
	Positive ^b^ (%)		n ^c^	Positive (%)		n	Positive (%)		n	
Allspice	×	0.0	3	⏺	33.3	3	×	0.0	3	[10]
Anise	×	0.0	3	×	0.0	3	×	0.0	3	[10]
Basil	×	0.0	2	×	0.0	2	×	0.0	2	[10]
Bay leaf	×	0.0	2	×	0.0	2	⏺	50.0	2	[10]
Caraway	×	0.0	2	×	0.0	2	⏺	100	2	[10]
Cardamom	×	0.0	4	×	0.0	4	⏺	75.0	4	[10]
Chili	○	14.3	7	⏺	42.9	7	⏺	100	7	[10]
Cinnamon	⏺	66.7	3	⏺	66.7	3	⏺	66.7	3	[10]
Cloves	⏺	50.0	2	×	0.0	2	⏺	50.0	2	[10]
Coriander	×	0.0	2	×	0.0	2	⏺	100	2	[10]
Cumin	×	0.0	5	×	0.0	5	⏺	100	5	[10]
Fennel	×	0.0	2	×	0.0	2	⏺	100	2	[10]
Fenugreek	×	0.0	4	×	0.0	4	⏺	50.0	4	[10]
Garlic	×	0.0	2	⏺	50.0	2	⏺	100	2	[10]
Ginger	⏺	33.3	3	⏺	33.3	3	⏺	66.7	3	[10]
Licorice	-	-	0	⏺	45.2	31	-	-	0	[141]
Marjoram	×	0.0	2	×	0.0	2	⏺	100	2	[10]
Mint	×	0.0	3	⏺	33.3	3	⏺	66.7	3	[10]
Nutmeg	×	0.0	2	⏺	50.0	2	⏺	50.0	2	[10]
Onion	×	0.0	4	⏺	50.0	4	⏺	50.0	4	[10]
Oregano	×	0.0	3	⏺	33.3	3	⏺	100	3	[10]
Paprika	○	5.9	34	⏺	61.8	34	⏺	100	34	[10,107]
Parsley	×	0.0	1	×	0.0	1	×	0.0	1	[10]
Pepper, black	×	0.0	4	○	25.0	4	⏺	75.0	4	[10]
Pepper, white	×	0.0	2	⏺	50.0	2	⏺	50.0	2	[10]
Rosemary	×	0.0	2	×	0.0	2	⏺	50.0	2	[10]
Sage	×	0.0	3	⏺	66.7	3	⏺	66.7	3	[10]
Sumac	×	0.0	2	⏺	50.0	2	⏺	100	2	[10]
Thyme	×	0.0	3	×	0.0	3	⏺	100	3	[10]
Turmeric	⏺	50.0	2	×	0.0	2	⏺	100	2	[10]

Notes: ^a^ ALT = Altenuene, AOH = Alternariol, TEA = Tenuazonic acid; ^b^ Positive = the percentage of positive samples; ^c^ n = the total number of samples related to mycotoxin and spice from all publications involved; × = none occurrence (0%), ☆ = rare occurrence (up to 5%), ○ = low occurrence (up to 25%), ⏺ = moderate occurrence (up to 50%); ⏺ = high occurrence (up to 75%), ⏺ = very high occurrence (more than 75%).

**Table 8:** Fungi: Natural occurrence of *Aspergillus, Penicillium* and *Fusarium* genera in spices in the last 5 years (since 2015).

Microfungi/Spice	*Aspergillus* spp.			*Penicillium* spp.			*Fusarium* spp.			Reference
	Positive ^a^ (%)		n ^b^	Positive (%)		n	Positive (%)		n	
Anise	⏺	100	3	⏺	100	2	⏺	50.0	2	[109,111,144]
Basil	⏺	100	1	⏺	100	1	×	0.0	1	[109]
Bay leaf	⏺	100	2	⏺	100	2	⏺	50.0	2	[109,145]
Caraway	⏺	100	6	⏺	80.0	5	○	20.0	5	[8,109,111,144,145,146]
Cardamom	⏺	83.3	6	⏺	50.0	6	⏺	33.3	6	[109,119,126,127,145,146]
Chili	⏺	100	15	⏺	66.7	9	⏺	100	6	[8,106,109,111,113,131,132,144,146,147,148,149,150,151]
Cinnamon	⏺	50.0	8	⏺	50.0	6	×	0.0	5	[109,111,127,131,144,145,146,149]
Cloves	⏺	37.5	8	○	14.3	7	×	0.0	4	[109,127,131,145,146,147,148,149]
Coriander	⏺	100	6	⏺	60.0	5	×	0.0	5	[8,109,111,144,145,146]
Cumin	⏺	80.0	5	⏺	75.0	4	⏺	50.0	4	[8,109,111,144,146]
Cumin, black	⏺	100	2	⏺	50.0	2	⏺	50.0	2	[109,145]
Curry	⏺	75.0	4	×	0.0	4	×	0.0	2	[144,146,147,148]
Fennel	⏺	100	8	⏺	50.0	6	⏺	60.0	5	[8,109,111,126,127,131,145,149]
Fenugreek	⏺	100	3	⏺	66.7	3	⏺	33.3	3	[8,109,146]
Garlic	⏺	100	3	×	0.0	3	×	0.0	1	[109,147,148]
Ginger	⏺	100	7	⏺	33.3	6	⏺	50.0	4	[8,109,111,144,146,147,148]
Licorice	⏺	100	1	⏺	100	1	×	0.0	1	[109]
Mace	⏺	100	1	⏺	100	1	⏺	100	1	[126]
Marjoram	⏺	100	1	⏺	100	1	×	0.0	1	[109]
Mint	⏺	100	1	⏺	100	1	×	0.0	1	[109]
Mustard	⏺	66.7	3	⏺	66.7	3	×	0.0	3	[109,127,146]
Nutmeg	⏺	90.0	10	⏺	60.0	10	×	0.0	4	[105,109,127,144,146,147,148,152,153,154]
Oregano	⏺	100	2	⏺	100	1	-	-	0	[131,149]
Paprika	⏺	100	2	⏺	100	1	⏺	100	1	[107,111]
Parsley	⏺	100	1	⏺	100	1	×	0.0	1	[109]
Pepper, black	⏺	91.7	12	⏺	75.0	8	⏺	33.3	6	[8,109,111,118,126,127,131,144,146,149,151,155]
Pepper, white	⏺	100	6	⏺	50.0	4	×	0.0	2	[118,131,144,146,149,151]
Rosemary	⏺	100	3	⏺	50.0	2	×	0.0	1	[109,131,149]
Saffron	⏺	66.7	3	⏺	50.0	2	×	0.0	2	[109,111,146]
Star anise	×	0.0	1	×	0.0	1	×	0.0	1	[127]
Sumac	⏺	50.0	2	×	0.0	2	⏺	50.0	2	[109,145]
Thyme	⏺	100	3	⏺	33.3	3	⏺	100	1	[109,147,148]
Turmeric	⏺	100	5	⏺	80.0	5	⏺	60.0	5	[8,109,126,144,146]

Notes: ^a^ Positive = the percentage of studies with at least one related spice sample positive on related mold; ^b^ n = number of studies concerning related spice and mold; × = none occurrence (0%); ☆ = rare occurrence (up to 5%); ○ = low occurrence (up to 25%); ⏺  = moderate occurrence (up to 50%); ⏺ = high occurrence (up to 75%); ⏺ = very high occurrence (more than 75%).

**Table 9:** Fungi: Natural occurrence of *Aspergillus* species in spices in the last 5 years (since 2015).

Microfungi/Spice	*A. flavus*			*A. parasiticus*			*A. niger*			*A. tamari*			*A. terreus*			*A. versicolor*			*A. ochraceus*			*A. carbonarius*			Reference
	Positive ^a^ (%)		n ^b^	Positive (%)		n	Positive (%)		n	Positive (%)		n	Positive (%)		n	Positive (%)		n	Positive (%)		n	Positive (%)		n	
Anise	×	0.0	1	×	0.0	1	⏺	100	1	⏺	50.0	2	×	0.0	1	×	0.0	1	⏺	100	1	-	-	0	[109,144]
Basil	×	0.0	1	×	0.0	1	⏺	100	1	×	0.0	1	×	0.0	1	⏺	100	1	⏺	100	1	-	-	0	[109]
Bay leaf	⏺	50.0	2	×	0.0	1	⏺	100	2	×	0.0	1	⏺	100	1	×	0.0	1	⏺	100	1	-	-	0	[109,145]
Caraway	⏺	33.3	3	×	0.0	3	⏺	100	4	×	0.0	3	⏺	50.0	2	×	0.0	2	⏺	50.0	2	-	-	0	[8,109,144,145,146]
Cardamom	⏺	83.3	6	⏺	50.0	4	⏺	100	6	×	0.0	1	⏺	33.3	3	⏺	50.0	2	⏺	66.7	3	-	-	0	[109,119,126,127,145,146]
Chili	⏺	90.0	10	⏺	55.6	9	⏺	90.9	11	⏺	80.0	5	⏺	50.0	4	⏺	75.0	4	⏺	40.0	5	⏺	50.0	4	[8,109,113,131,132,144,146,147,148,149,150,151]
Cinnamon	⏺	33.3	6	○	25.0	4	⏺	66.7	6	⏺	33.3	3	×	0.0	2	×	0.0	2	⏺	66.7	3	×	0.0	2	[109,127,131,144,145,146,149]
Cloves	⏺	37.5	8	⏺	33.3	6	○	25.0	8	×	0.0	2	×	0.0	2	×	0.0	3	×	0.0	3	×	0.0	2	[109,127,131,145,146,147,148,149]
Coriander	⏺	33.3	3	⏺	33.3	3	⏺	75.0	4	×	0.0	3	×	0.0	2	×	0.0	2	×	0.0	2	-	-	0	[8,109,144,145,146]
Cumin	⏺	50.0	2	×	0.0	3	⏺	66.7	3	⏺	33.3	3	⏺	50.0	2	⏺	50.0	2	⏺	100	2	-	-	0	[8,109,144,146]
Cumin, black	⏺	50.0	2	×	0.0	1	⏺	100	2	×	0.0	1	×	0.0	1	⏺	100	1	×	0.0	1	-	-	0	[109,145]
Curry	⏺	33.3	3	⏺	33.3	3	⏺	33.3	3	⏺	100	1	-	-	0	×	0.0	1	-	-	0	-	-	0	[144,146,147,148]
Fennel	⏺	66.7	6	○	20.0	5	⏺	85.7	7	×	0.0	3	⏺	75.0	4	○	25.0	4	⏺	40.0	5	×	0.0	2	[8,109,126,127,131,145,149]
Fenugreek	⏺	50.0	2	×	0.0	3	⏺	100	3	×	0.0	2	×	0.0	2	×	0.0	2	⏺	50.0	2	-	-	0	[8,109,146]
Garlic	⏺	33.3	3	⏺	66.7	3	⏺	66.7	3	×	0.0	1	×	0.0	1	⏺	50.0	2	×	0.0	1	-	-	0	[109,147,148]
Ginger	⏺	75.0	4	⏺	60.0	5	⏺	60.0	5	×	0.0	3	⏺	50.0	2	⏺	33.3	3	⏺	50.0	2	-	-	0	[8,109,144,146,147,148]
Licorice	⏺	100	1	×	0.0	1	⏺	100	1	⏺	100	1	×	0.0	1	×	0.0	1	×	0.0	1	-	-	0	[109]
Mace	⏺	100	1	⏺	100	1	⏺	100	1	-	-	0	×	0.0	1	⏺	100	1	×	0.0	1	-	-	0	[126]
Marjoram	×	0.0	1	×	0.0	1	⏺	100	1	×	0.0	1	×	0.0	1	×	0.0	1	×	0.0	1	-	-	0	[109]
Mint	×	0.0	1	×	0.0	1	⏺	100	1	×	0.0	1	×	0.0	1	⏺	100	1	×	0.0	1	-	-	0	[109]
Mustard	×	0.0	3	⏺	50.0	2	⏺	33.3	3	×	0.0	1	×	0.0	1	×	0.0	1	×	0.0	1	-	-	0	[109,127,146]
Nutmeg	⏺	66.7	9	⏺	75.0	4	⏺	55.6	9	⏺	60.0	5	×	0.0	1	⏺	50.0	4	⏺	66.7	3	-	-	0	[105,109,127,144,146,147,148,152,153,154]
Oregano	⏺	100	2	×	0.0	2	⏺	100	2	⏺	100	1	⏺	100	1	⏺	100	1	⏺	100	2	×	0.0	2	[131,149]
Parsley	×	0.0	1	×	0.0	1	⏺	100	1	⏺	100	1	×	0.0	1	×	0.0	1	⏺	100	1	-	-	0	[109]
Pepper, black	⏺	88.9	9	⏺	75.0	8	⏺	88.9	9	⏺	50.0	4	⏺	50.0	4	⏺	100	4	⏺	100	5	⏺	100	2	[8,109,118,126,127,131,144,146,149,151,155]
Pepper, white	⏺	80.0	5	⏺	75.0	4	⏺	100	4	⏺	100	2	⏺	100	1	⏺	100	1	×	0.0	2	×	0.0	2	[118,131,144,146,149,151]
Rosemary	⏺	66.7	3	⏺	66.7	3	⏺	100	3	×	0.0	2	×	0.0	2	⏺	50.0	2	×	0.0	3	×	0.0	2	[109,131,149]
Saffron	×	0.0	2	×	0.0	2	⏺	50.0	2	×	0.0	1	×	0.0	1	⏺	100	1	×	0.0	1	-	-	0	[109,146]
Star anise	×	0.0	1	-	-	0	×	0.0	1	-	-	0	-	-	0	-	-	0	-	-	0	-	-	0	[127]
Sumac	⏺	50.0	2	×	0.0	1	×	0.0	2	×	0.0	1	×	0.0	1	×	0.0	1	×	0.0	1	-	-	0	[109,145]
Thyme	⏺	100	3	⏺	66.7	3	⏺	33.3	3	×	0.0	1	×	0.0	1	⏺	50.0	2	⏺	100	1	-	-	0	[109,147,148]
Turmeric	⏺	33.3	3	⏺	50.0	4	⏺	50.0	4	×	0.0	3	×	0.0	3	×	0.0	3	⏺	100	3	-	-	0	[8,109,126,144,146]

Notes: ^a^ Positive = the percentage of studies with at least one related spice sample positive on related mold; ^b^ n = number of studies concerning related spice and mold; × = none occurrence (0%); ☆ = rare occurrence (up to 5%); ○ = low occurrence (up to 25%); ⏺ = moderate occurrence (up to 50%); ⏺ = high occurrence (up to 75%); ⏺ = very high occurrence (more than 75%)

**Table 10:** Fungi: Natural occurrence of *Penicillium, Fusarium, Alternaria*, and *Rhizopus* species in spices in the last 5 years (since 2015).

Microfungi/Spice	*Penicillium citrinum*			*Penicillium verrucosum*			*Fusarium verticillioides*			*Alternaria alternata*			*Rhizopus nigricans*			*Rhizopus oryzae*			Reference
	Positive ^a^ (%)		n ^b^	Positive (%)		n	Positive (%)		n	Positive (%)		n	Positive (%)		n	Positive (%)		n	
Anise	×	0.0	1	-	-	0	×	0.0	1	⏺	100	1	×	0.0	1	-	-	0	[109]
Basil	×	0.0	1	-	-	0	×	0.0	1	×	0.0	1	×	0.0	1	-	-	0	[109]
Bay leaf	×	0.0	1	-	-	0	×	0.0	1	×	0.0	2	×	0.0	1	⏺	100	1	[109,145]
Caraway	⏺	50.0	2	×	0.0	1	×	0.0	2	×	0.0	3	×	0.0	2	×	0.0	2	[8,109,145]
Cardamom	⏺	50.0	4	⏺	100	2	⏺	50.0	2	⏺	66.7	3	×	0.0	2	⏺	100	3	[109,119,126,127,145]
Chili	⏺	33.3	6	⏺	100	1	⏺	100	2	⏺	66.7	3	×	0.0	2	⏺	100	1	[8,109,132,147,148,149]
Cinnamon	×	0.0	3	-	-	0	×	0.0	1	×	0.0	2	×	0.0	1	×	0.0	1	[109,127,145,149]
Cloves	×	0.0	5	-	-	0	×	0.0	1	×	0.0	2	×	0.0	1	×	0.0	1	[109,127,145,147,148,149]
Coriander	⏺	50.0	2	⏺	100	1	×	0.0	2	⏺	33.3	3	⏺	50.0	2	⏺	50.0	2	[8,109,145]
Cumin	⏺	50.0	2	×	0.0	1	×	0.0	2	⏺	50.0	2	⏺	100	2	⏺	100	1	[8,109]
Cumin, black	×	0.0	1	-	-	0	×	0.0	1	×	0.0	2	×	0.0	1	×	0.0	1	[109,145]
Curry	×	0.0	2	-	-	0	-	-	0	-	-	0	-	-	0	-	-	0	[147,148]
Fennel	○	20.0	5	×	0.0	2	⏺	66.7	3	×	0.0	3	×	0.0	3	⏺	66.7	3	[8,109,126,127,145,149]
Fenugreek	⏺	50.0	2	⏺	100	1	⏺	50.0	2	⏺	50.0	2	×	0.0	2	⏺	100	1	[8,109]
Garlic	×	0.0	3	-	-	0	×	0.0	1	×	0.0	1	×	0.0	1	-	-	0	[109,147,148]
Ginger	○	25.0	4	⏺	100	1	⏺	50.0	2	×	0.0	2	×	0.0	2	⏺	100	1	[8,109,147,148]
Licorice	×	0.0	1	-	-	0	×	0.0	1	×	0.0	1	×	0.0	1	-	-	0	[109]
Mace	⏺	100	1	⏺	100	1	⏺	100	1	-	-	0	⏺	100	1	⏺	100	1	[126]
Marjoram	⏺	100	1	-	-	0	×	0.0	1	×	0.0	1	×	0.0	1	-	-	0	[109]
Mint	×	0.0	1	-	-	0	×	0.0	1	×	0.0	1	×	0.0	1	-	-	0	[109]
Mustard	⏺	50.0	2	-	-	0	×	0.0	1	⏺	100	1	⏺	100	1	-	-	0	[109,127]
Nutmeg	⏺	50.0	8	-	-	0	×	0.0	1	×	0.0	1	×	0.0	1	-	-	0	[105,109,127,147,148,152,153,154]
Oregano	×	0.0	1	-	-	0	-	-	0	-	-	0	-	-	0	-	-	0	[131,149]
Parsley	×	0.0	1	-	-	0	×	0.0	1	×	0.0	1	×	0.0	1	-	-	0	[109]
Pepper, black	⏺	40.0	5	⏺	100	2	⏺	66.7	3	×	0.0	2	⏺	66.7	3	⏺	100	2	[8,109,126,127,149]
Pepper, white	⏺	100	1	-	-	0	-	-	0	-	-	0	-	-	0	-	-	0	[149]
Rosemary	⏺	50.0	2	-	-	0	×	0.0	1	×	0.0	1	×	0.0	1	-	-	0	[109,149]
Saffron	×	0.0	1	-	-	0	×	0.0	1	×	0.0	1	×	0.0	1	-	-	0	[109]
Star anise	×	0.0	1	-	-	0	-	-	0	-	-	0	-	-	0	-	-	0	[127]
Sumac	×	0.0	1	-	-	0	×	0.0	1	×	0.0	2	×	0.0	1	⏺	100	1	[109,145]
Thyme	×	0.0	3	-	-	0	⏺	100	1	×	0.0	1	×	0.0	1	-	-	0	[109,147,148]
Turmeric	⏺	100	3	⏺	100	2	⏺	66.7	3	×	0.0	2	×	0.0	3	⏺	50.0	2	[8,109,126]

Notes: ^a^ Positive = the percentage of studies with at least one related spice sample positive on related mold; ^b^ n = number of studies concerning related spice and mold; × = none occurrence (0%); ☆ = rare occurrence (up to 5%); ○ = low occurrence (up to 25%); ⏺ = moderate occurrence (up to 50%); ⏺ = high occurrence (up to 75%); **⏺** = very high occurrence (more than 75%).

**Table 11:** Summary of studies in which above-the-limit values of mycotoxins have been recorded in relation to the European Union legislation.

Mycotoxin/Spice	AFB_1_						Total AFs						OTA						Reference
	Positive		Over MPL ^a^			n_T_^c^	Positive		Over MPL			n_T_	Positive		Over MPL			n_T_	
	%	n ^b^	%		n ^b^		%	n	%		n		%	n	%		n		
Pepper, black	58.3	7	⏺	41.7	5	12	60.0	6	⏺	30.0	3	10	66.7	8	○	25.0	3	12	[8,9,35,108,109,110,111,112,116,117,118,120,121,122,123,125,126,127,129,130,131]
Pepper, white	33.3	1	×	0.0	0	3	50.0	2	○	25.0	1	4	25.0	1	×	0.0	0	4	[9,35,112,125,131]
Nutmeg	33.3	2	○	16.7	1	6	75.0	3	⏺	75.0	3	4	100	3	⏺	66.7	2	3	[9,105,109,120,123,127,135,152,153]
Ginger	100	7	⏺	42.9	3	7	66.7	4	⏺	33.3	2	6	83.3	5	○	16.7	1	6	[8,9,109,110,111,117,120,122,130,139,140,148]
Turmeric	83.3	5	⏺	50.0	3	6	75.0	6	⏺	37.5	3	8	100	5	⏺	40.0	2	5	[8,9,109,110,112,116,121,122,125,126]
Chili	94.4	17	⏺	72.2	13	18	91.7	11	⏺	83.3	10	12	83.3	10	⏺	50.0	6	12	[8,9,106,108,110,111,112,113,114,115,116,117,118,120,121,122,128,129,130,131,132,133,135,136]
Paprika	100	3	⏺	66.7	2	3	100	3	⏺	100	3	3	100	4	⏺	50.0	2	4	[9,107,111,120,133]
Licorice	50.0	1	no MPL			2	50.0	1	no MPL			2	100	2	⏺	50.0	1	2	[109,120,141]

Notes: ^a^ MPL = maximum permissible limit; ^b^ n = number of studies; ^c^ n_T_ = total number of publications related to mycotoxins in spice, with mean or maximum value available or with no mycotoxin occurrence; × = none over-MPL occurrence (0%); ☆ = rare over-MPL occurrence (up to 5%); ○ = low over-MPL occurrence (up to 25%); ⏺ = moderate over-MPL occurrence (up to 50%); ⏺ = high over-MPL occurrence (up to 75%); ⏺ = very high over-MPL occurrence (more than 75%).

**Table 12:** Some of the highest values of aflatoxin B_1_ and total aflatoxins contamination in spices, based on the RASFF database in 2015–2019.

No.	Origin	Spice	Maximum Level of AFB_1_ (µg/kg)	Maximum Level of Total AFs ^a^ (µg/kg)	Classification ^b^	Date of Case
**1**	Nigeria	Suya pepper	300.00	360.00	I	15/02/2017
**2**	Indonesia, Sri Lanka ^c^	Nutmeg	180.00	210.60	BR, BR ^c^	21/09/2015, 27/01/2016 ^c^
**3**	Malawi	Chilies	96.20	116.00	A	29/08/2017
**4**	Ghana, Ghana ^c^	Kebab spice	93.40	112.30	A, BR ^c^	14/07/2015, 12/10/2016 ^c^
**5**	Ethiopia	Paprika powder	73.44	239.57	BR	19/01/2016
**6**	Ethiopia	Berbere spice	35.00	91.00	BR	13/05/2016
**7**	Sri Lanka	Curry powder	34.30	36.50	A	25/01/2018
**8**	Netherlands	White pepper	23.90	54.70	A	09/12/2015
**9**	Nigeria	Ginger	22.70	48.70	A	06/04/2017
**10**	India	Turmeric powder	14.80	16.30	A	05/01/2017
**11**	India	Cayenne pepper	11.10	11.60	BR	19/07/2019
**12**	India	Ground cumin	8.82	12.19	A	02/08/2019

Notes: ^a^ Total AFs = sum of aflatoxins B_1_, B_2_, G_1,_ and G_2_; ^b^ I = Information, BR = Border rejection, A = alert; ^c^ comma-separated data correspond to value of AFB_1_ and total AFs, respectively, in case of data originated from separate notifications. Processed according to RASFF [156].

## Supplementary Materials

The following are available online at https://www.mdpi.com/2072-6651/12/12/789/s1. Table S1: Studies positivity: Natural occurrence of mycotoxins produced by *Aspergillus* and *Penicillium* species in spices in the last 5 years (since 2015). Table S2: Studies positivity: Natural occurrence of *Fusarium* mycotoxins in spices in the last 5 years (since 2015). Table S3: Studies positivity: Natural occurrence of *Alternaria* mycotoxins in spices in the last 5 years (since 2015).
