# Supplementary material for: A Review on Mycotoxins and Microfungi in Spices in the Light of the Last Five Years
Source: Toxins (Basel). 2020 Dec 11;12(12):789. doi: 10.3390/toxins12120789 (PMC7763258; doi:10.3390/toxins12120789)
Supplement: Supplementary file 1 [file toxins-12-00789-s001.pdf]

Supplementary Materials: A Review on Mycotoxins and Microfungi in Spices in the Light of the Last Five Years

Darina Pickova, Vladimir Ostry, Jan Malir, Jakub Toman and Frantisek Malir

Table S1. Studies positivity: Natural occurrence of mycotoxins produced by *Aspergillus* and *Penicillium* species in spices in the last 5 years (since 2015).

Mycotoxin[a]/ Spice	AFB_1 Positive[b] (%)	n[c]	AFB_2 Positive (%)	n	AFG_1 Positive (%)	n	AFG_2 Positive (%)	n	AFs Positive (%)	n	OTA Positive (%)	n	CIT Positive (%)	n	Reference
Allspice	- -	0	- -	0	- -	0	- -	0	● 100	1	× 0.0	1	- -	0	[9]
Anise	● 100	2	× 0.0	1	● 100	1	× 0.0	1	● 75.0	4	● 100	1	- -	0	[9,109,111,122]
Basil	× 0.0	2	- -	0	- -	0	- -	0	× 0.0	1	× 0.0	2	× 0.0	1	[9,110,123]
Bay leaf	× 0.0	3	× 0.0	2	● 50.0	2	● 50.0	2	● 50.0	4	× 0.0	1	- -	0	[9,110,122,124]
Caraway	● 40.0	5	● 50.0	4	○ 25.0	4	○ 25.0	4	● 50.0	6	● 66.7	3	× 0.0	1	[8,9,120,124–126]
Cardamom	× 0.0	2	× 0.0	1	× 0.0	1	× 0.0	1	● 60.0	5	● 66.7	3	- -	0	[9,109,119,122,126,127]
Carom	● 100	1	× 0.0	1	× 0.0	1	× 0.0	1	● 100	1	- -	0	- -	0	[125]
Chili	● 95.7	23	● 75.0	12	● 50.0	12	● 45.5	11	● 96.3	27	● 85.7	14	● 100	2	[8,9,106,108,110–118,120–122,128–138,147,148]
Cinnamon	● 50.0	8	○ 20.0	5	● 40.0	5	○ 20.0	5	● 57.1	7	● 33.3	3	- -	0	[9,110–112,116,121,122,125,127,131]
Cloves	× 0.0	2	× 0.0	1	× 0.0	1	× 0.0	1	● 40.0	5	× 0.0	3	- -	0	[9,35,122,127,131,147,148]
Coriander	● 57.1	7	● 50.0	6	● 33.3	6	● 33.3	6	● 62.5	8	● 75.0	4	● 100	1	[8,9,109,111,112,120,124,125]
Cumin	● 57.1	7	● 40.0	5	● 60.0	5	● 40.0	5	● 66.7	6	● 66.7	3	● 100	1	[8,9,109–112,122,125]
Cumin, black	● 66.7	3	● 50.0	2	● 100	2	● 50.0	2	● 100	2	- -	0	- -	0	[109,110,125]
Curry	● 100	1	● 100	1	● 100	1	● 100	1	● 33.3	3	● 100	1	- -	0	[112,147,148]
Dawadawa	● 100	1	- -	0	- -	0	- -	0	● 100	1	● 100	1	- -	0	[130]
Fennel	● 40.0	10	● 28.6	7	● 42.9	7	● 28.6	7	● 60.0	10	● 60.0	5	× 0.0	1	[8,9,109–112,124–127,131]
Fenugreek	● 50.0	2	● 50.0	2	● 100	2	● 50.0	2	● 100	3	● 50.0	2	● 100	1	[8,9,109]
Garlic	- -	0	- -	0	- -	0	- -	0	× 0.0	3	● 100	1	- -	0	[9,147,148]
Ginger	● 100	8	● 60.0	5	● 40.0	5	● 40.0	5	● 81.8	11	● 83.3	6	● 100	1	[8,9,109–111,117,120,122,130,139,140,147,148]
Licorice	● 50.0	2	× 0.0	2	● 50.0	2	× 0.0	2	● 50.0	2	● 100	2	● 100	1	[109,120,141]
Mace	- -	0	- -	0	- -	0	- -	0	● 100	1	● 100	1	- -	0	[126]
Marjoram	● 100	1	× 0.0	1	× 0.0	1	× 0.0	1	● 50.0	2	● 100	1	- -	0	[9,109]
Mint	× 0.0	3	× 0.0	2	× 0.0	2	× 0.0	2	× 0.0	3	× 0.0	1	- -	0	[9,110,124]
Mustard	● 50.0	2	× 0.0	1	● 100	1	● 100	1	● 100	1	● 100	1	- -	0	[109,120,127]
Nutmeg	● 33.3	6	● 50.0	2	● 50.0	2	× 0.0	2	● 85.7	7	● 100	3	× 0.0	1	[9,105,109,120,123,127,135,147,148,152,153]
Onion	× 0.0	1	- -	0	× 0.0	1	- -	0	× 0.0	2	× 0.0	2	- -	0	[9,133]

Mycotoxin[a]/ Spice	AFB$_1$ Positive[b] (%)	n[c]	AFB$_2$ Positive (%)	n	AFG$_1$ Positive (%)	n	AFG$_2$ Positive (%)	n	AFs Positive (%)	n	OTA Positive (%)	n	CIT Positive (%)	n	Reference
Oregano	× 0.0	4	× 0.0	3	× 0.0	3	× 0.0	3	○ 25.0	4	× 0.0	3	× 0.0	1	[9,123,124,131]
Paprika	● 100	3	● 100	1	● 100	2	● 100	1	● 100	4	● 100	4	- -	0	[9,107,111,120,133]
Parsley	● 100	1	● 100	1	● 100	1	× 0.0	1	◔ 50.0	2	× 0.0	1	- -	0	[9,109]
Pepper, black	◑ 64.3	14	◑ 57.1	7	◔ 42.9	7	◔ 28.6	7	◑ 73.3	15	◑ 66.7	12	◑ 66.7	3	[8,9,35,108–112,116–118,120–123,125–127,129–131,134]
Pepper, white	◔ 33.3	3	◔ 33.3	3	◔ 33.3	3	◔ 33.3	3	◔ 50.0	4	○ 25.0	4	- -	0	[9,35,112,125,131]
Rosemary	◔ 50.0	4	◔ 50.0	4	○ 25.0	4	◔ 50.0	4	● 100	5	◔ 50.0	2	- -	0	[9,109,124,131]
Saffron	● 100	1	- -	0	- -	0	- -	0	◔ 50.0	2	× 0.0	1	- -	0	[9,111]
Sage	◔ 50.0	2	× 0.0	1	● 100	1	× 0.0	1	● 100	3	● 100	1	- -	0	[9,109,110]
Star anise	× 0.0	1	- -	0	- -	0	- -	0	- -	0	- -	0	- -	0	[127]
Sumac	× 0.0	1	- -	0	- -	0	- -	0	× 0.0	1	× 0.0	1	- -	0	[9,110]
Thyme	◔ 40.0	5	× 0.0	3	◔ 33.3	3	× 0.0	3	◔ 42.9	7	× 0.0	2	× 0.0	1	[9,109,110,123,124,147,148]
Turmeric	● 85.7	7	◑ 66.7	6	◑ 66.7	6	○ 16.7	6	● 77.8	9	● 100	5	× 0.0	1	[8,9,109,110,112,116,121,122,125,126]

Notes: [a] AFB$_1$ = Aflatoxin B$_1$, AFB$_2$ = Aflatoxin B$_2$, AFG$_1$ = Aflatoxin G$_1$, AFG$_2$ = Aflatoxin G$_2$, AFs = Aflatoxins, OTA = Ochratoxin A, CIT = Citrinin; [b] Positive = the percentage of studies with at least one related spice sample positive on related mycotoxin; [c] n = number of studies concerning related spice and mycotoxin; × = none occurrence (0 %); ☆ = rare occurrence (up to 5 %); ○ = low occurrence (up to 25 %); ◔ = moderate occurrence (up to 50 %); ◑ = high occurrence (up to 75 %); ● = very high occurrence (more than 75 %).

Table S2. Studies positivity: Natural occurrence of *Fusarium* mycotoxins in spices in the last 5 years (since 2015).

Mycotoxin[a]/ Spice	FB1 Positive[b] (%)		n[c]	FB2 Positive (%)		n	DON Positive (%)		n	NIV Positive (%)		n	T-2 Positive (%)		n	HT-2 Positive (%)		n	ZEA Positive (%)		n	Reference
Basil	×	0.0	2	×	0.0	1	×	0.0	1	×	0.0	1	×	0.0	1	×	0.0	1	×	0.0	1	[123,143]
Bay leaf	×	0.0	3	×	0.0	1	-	-	0	-	-	0	×	0.0	2	×	0.0	2	-	-	0	[124,143]
Caraway	×	0.0	2	-	-	0	-	-	0	-	-	0	×	0.0	2	×	0.0	2	-	-	0	[124]
Chili	×	0.0	1	●	100	2	-	-	0	-	-	0	-	-	0	-	-	0	×	0.0	1	[118,133,137]
Coriander	×	0.0	3	×	0.0	1	-	-	0	-	-	0	◍	50.0	2	×	0.0	2	-	-	0	[124,143]
Dawadawa	●	100	1	●	100	1	×	0.0	1	×	0.0	1	●	100	1	●	100	1	●	100	1	[34]
Fennel	×	0.0	2	-	-	0	-	-	0	-	-	0	×	0.0	2	×	0.0	2	-	-	0	[124]
Garlic	●	100	1	×	0.0	1	-	-	0	-	-	0	-	-	0	-	-	0	-	-	0	[142]
Licorice	●	100	1	×	0.0	1	●	100	1	-	-	0	×	0.0	1	-	-	0	●	100	1	[141]
Mint	◍	33.3	3	×	0.0	1	-	-	0	-	-	0	◍	50.0	2	×	0.0	2	-	-	0	[124,143]
Nutmeg	●	100	1	-	-	0	×	0.0	1	×	0.0	1	×	0.0	1	×	0.0	1	×	0.0	1	[123]
Onion	●	100	1	●	100	1	-	-	0	-	-	0	-	-	0	-	-	0	-	-	0	[133]
Oregano	×	0.0	3	-	-	0	×	0.0	1	×	0.0	1	×	0.0	3	×	0.0	3	×	0.0	1	[123,124]
Paprika	◍	50.0	2	●	100	2	●	100	1	●	100	1	●	100	1	●	100	1	●	100	1	[107,133]
Pepper, black	◍	50.0	2	-	-	0	×	0.0	1	×	0.0	1	×	0.0	1	×	0.0	1	×	0.0	1	[118,123]
Rosemary	×	0.0	2	-	-	0	-	-	0	-	-	0	×	0.0	2	×	0.0	2	-	-	0	[124]
Thyme	○	25.0	4	×	0.0	1	●	100	1	×	0.0	1	×	0.0	3	×	0.0	3	●	100	1	[123,124,143]

Notes: [a] FB_1 = fumonisin B_1, FB_2 = Fumonisin B_2, DON = Deoxynivalenol, NIV = Nivalenol, T-2 = T-2 toxin, HT-2 = HT-2 toxin, ZEA = Zearalenone; [b] Positive = the percentage of studies with at least one related spice sample positive on related mycotoxin; [c] n = number of studies concerning related spice and mycotoxin; × = none occurrence (0 %); ☆ = rare occurrence (up to 5 %); ○ = low occurrence (up to 25 %); ◍ = moderate occurrence (up to 50 %); ◉ = high occurrence (up to 75 %); ● = very high occurrence (more than 75 %).

Table S3. Studies positivity: Natural occurrence of *Alternaria* mycotoxins in spices in the last 5 years (since 2015).

Mycotoxin[a]/ Spice	ALT Positive[b] (%)		n[c]	AOH Positive (%)		n	TEA Positive (%)		n	Reference
Allspice	×	0.0	1	●	100	1	×	0.0	1	[10]
Anise	×	0.0	1	×	0.0	1	×	0.0	1	[10]
Basil	×	0.0	1	×	0.0	1	×	0.0	1	[10]
Bay leaf	×	0.0	1	×	0.0	1	●	100	1	[10]
Caraway	×	0.0	1	×	0.0	1	●	100	1	[10]
Cardamom	×	0.0	1	×	0.0	1	●	100	1	[10]
Chili	●	100	1	●	100	1	●	100	1	[10]
Cinnamon	●	100	1	●	100	1	●	100	1	[10]
Cloves	●	100	1	×	0.0	1	●	100	1	[10]
Coriander	×	0.0	1	×	0.0	1	●	100	1	[10]
Cumin	×	0.0	1	×	0.0	1	●	100	1	[10]
Fennel	×	0.0	1	×	0.0	1	●	100	1	[10]
Fenugreek	×	0.0	1	×	0.0	1	●	100	1	[10]
Garlic	×	0.0	1	●	100	1	●	100	1	[10]
Ginger	●	100	1	●	100	1	●	100	1	[10]
Licorice	-	-	0	●	100	1	-	-	0	[141]
Marjoram	×	0.0	1	×	0.0	1	●	100	1	[10]
Mint	×	0.0	1	●	100	1	●	100	1	[10]
Nutmeg	×	0.0	1	●	100	1	●	100	1	[10]
Onion	×	0.0	1	●	100	1	●	100	1	[10]
Oregano	×	0.0	1	●	100	1	●	100	1	[10]
Paprika	●	100	2	●	100	2	●	100	2	[10,107]
Parsley	×	0.0	1	×	0.0	1	×	0.0	1	[10]
Pepper, black	×	0.0	1	●	100	1	●	100	1	[10]
Pepper, white	×	0.0	1	●	100	1	●	100	1	[10]
Rosemary	×	0.0	1	×	0.0	1	●	100	1	[10]
Sage	×	0.0	1	●	100	1	●	100	1	[10]
Sumac	×	0.0	1	●	100	1	●	100	1	[10]
Thyme	×	0.0	1	×	0.0	1	●	100	1	[10]
Turmeric	●	100	1	×	0.0	1	●	100	1	[10]

Notes: [a] ALT = Altenuene, AOH = Alternariol, TEA = Tenuazonic acid; [b] Positive = the percentage of studies with at least one related spice sample positive on related mycotoxin; [c] n = a total number of studies concerning related spice and mycotoxin; × = none occurrence (0 %); ☆ = rare occurrence (up to 5 %); ○ = low occurrence (up to 25 %); ● (light grey) = moderate occurrence (up to 50 %); ● (grey) = high occurrence (up to 75 %); ● = very high occurrence (more than 75 %).
